# Mesomorphic Behavior in Silver(I) *N*-(4-Pyridyl) Benzamide with Aromatic π–π Stacking Counterions

**DOI:** 10.3390/ma11091666

**Published:** 2018-09-09

**Authors:** Issac Torres, Mauro Ruiz, Hung Phan, Noemi Dominguez, Jacobo Garcia, Thuc-Quyen Nguyen, Hayden Evans, Marino J. Resendiz, Tunna Baruah, Alejandro Metta, Atta Arif, Juan C. Noveron

**Affiliations:** 1 Department of Chemistry, University of Texas at El Paso, El Paso, TX 79968, USA; igtorres@miners.utep.edu (I.T.); mjruiz@miners.utep.edu (M.R.); ndominguez6@miners.utep.edu (N.D.); jegarcia1524@gmail.com (J.G.); ajmetta@utep.edu (A.M.); 2 Department of Chemistry & Biochemistry, University of California Santa Barbara, Santa Barbara, CA 93106-9510, USA; hphan@chem.ucsb.edu (H.P.); quyen@chem.ucsb.edu (T.-Q.N.); hevans@chem.ucsb.edu (H.E.); 3 Department of Chemistry, University of Colorado, Denver, CO 80217-3364, USA; MARINO.RESENDIZ@UCDENVER.EDU; 4 Department of Physics, University of Texas at El Paso, El Paso, TX 79968, USA; tbaruah@utep.edu; 5 Department of Chemistry, University of Utah, Salt Lake City, UT 84112, USA; arif@chemistry.utah.edu

**Keywords:** mesomorphic materials, metallo-mesogens, silver complexes, π–π stacking, crystalline solids

## Abstract

Organic semiconductor materials composed of π–π stacking aromatic compounds have been under intense investigation for their potential uses in flexible electronics and other advanced technologies. Herein we report a new family of seven π–π stacking compounds of silver(I) bis-*N*-(4-pyridyl) benzamide with varying counterions, namely [Ag(NPBA)2]X, where NPBA is *N*-(4-pyridyl) benzamine, X = NO_3_^−^ (**1**), ClO_4_^−^ (**2**), CF_3_SO_3_^−^ (**3**), PF_6_^−^ (**4**), BF_4_^−^ (**5**), CH_3_PhSO_3_^−^ (**6**), and PhSO_3_^−^ (**7**), which form extended π−π stacking networks in one-dimensional (1D), 2D and 3D directions in the crystalline solid-state via the phenyl moiety, with average inter-ring distances of 3.823 Å. Interestingly, the counterions that contain π–π stacking-capable groups, such as in **6** and **7**, can induce the formation of mesomorphic phases at 130 °C in dimethylformamide (DMF), and can generate highly branched networks at the mesoscale. Atomic force microscopy studies showed that 2D interconnected fibers form right after nucleation, and they extend from ~30 nm in diameter grow to reach the micron scale, which suggests that it may be possible to stop the process in order to obtain nanofibers. Differential scanning calorimetry studies showed no remarkable thermal behavior in the complexes in the solid state, which suggests that the mesomorphic phases originate from the mechanisms that occur in the DMF solution at high temperatures. An all-electron level simulation of the band gaps using NRLMOL (Naval Research Laboratory Molecular Research Library) on the crystals gave 3.25 eV for (**1**), 3.68 eV for (**2**), 1.48 eV for (**3**), 5.08 eV for (**4**), 1.53 eV for (**5**), and 3.55 eV for (**6**). Mesomorphic behavior in materials containing π–π stacking aromatic interactions that also exhibit low-band gap properties may pave the way to a new generation of highly branched organic semiconductors.

## 1. Introduction

Organic semiconductor materials composed of π–π stacking aromatic compounds have been under intense investigation for their potential uses in flexible electronics [1], field-effect transistors [2,3], light-emitting diodes [4,5,6], and photovoltaics [7,8,9]. In organic semiconductors, the electrical charge is transported along the direction of π–π stacking, and this is highly dependent on the surface area and morphology of the material. Due to this phenomenon, architectures that elucidate π–π stacking interactions and that can induce new morphologies are warranted. Engineering new materials with directional non-covalent interactions is a rapidly growing area [10,11,12,13], with the potential to generate novel molecular architectures using hydrogen bonding [14], metal–ligand coordination [15,16], and π–π stacking interactions [17,18,19,20,21,22]. In particular, aromatic π–π stacking interactions can direct the formation of one-dimensional (1D), 2D, and 3D crystalline nanostructures through self-assembly [23,24] and they can serve to engineer the physical properties of organic semiconductors [25,26,27,28,29]. With regard to intermolecular interactions, other factors such as solvents [30,31,32,33,34] and counterions [35,36,37,38,39,40,41,42,43,44,45] have been shown to play key roles in the structure of the final assembly. Therefore, the studies that establish the relationship of supramolecular structures in π–π stacking architectures can lead to a roadmap of synergistic intermolecular effects that pave the way to the rational design of materials.

Silver (Ag)(I) complexes have been reported to form molecular materials with an assorted set of structural topologies suitable for systematic structural studies [46,47]. For example, variations of the counterions in silver complexes have revealed trends useful in catalysis [48,49], optics [50], and medicine [50,51]. The anion influence in the assembly of silver-complexes is particularly notable [52,53,54,55,56,57], since the low coordination number and cationic nature of Ag(I) facilitates the influence of anions during self-organization in the crystalline solid-state. Generally, the anions act either as coordinated, bridging, or non-coordinated void-filling species and their influence on self-assembly is unpredictable.

Herein we carry out a systematic study of the influence of counterions during the formation of non-covalent networks in the crystalline solid state of silver(I) complexes of *N*-(4-pyridyl) benzamine (NPBA), namely [Ag(NPBA)_2_]X, where X = NO_3_^−^ (**1**), ClO_4_^−^ (**2**), CF_3_SO_3_^−^ (**3**), PF_6_^−^ (**4**), BF_4_^−^ (**5**), CH_3_C_6_H_4_SO_3_^−^ (**6**), and C_6_H_6_SO_3_^−^ (**7**). This allowed us to correlate supramolecular structures, resulting in π–π stacking networks of 1D, 2D, and 3D formations in the crystalline solid-state. This study allowed us to discover that π–π stacking capable counterions, such as those in **6** and **7**, form mesomorphic phases at 130 °C in dimethylformamide (DMF), giving rise to highly branched flexible fibers as small as ~30 nm in diameter and extended into the micron scale. This mesogenic property induced by π–π stacking capable counterions, will have important implications in the design of nano- and micro-structured organic semiconductors.

## 2. Experimental Section

Materials: Silver nitrate (Ag(NO)_3_ ≥ 99%), silver perchlorate (AgClO_4_ 97%), silver trifluoromethanesulfonate (CF_3_SO_3_Ag ≥ 99%), silver hexafluorophosphate (AgPF_6_ 98%), silver tetrafluoroborate (AgBF_4_ 98%), silver *p*-toluenesulfonate (CH_3_C_6_H4SO_3_Ag ≥ 99%), *N*,*N*-dimethylformamide (HCON(CH_3_)_2_ 99.8%), 1-propanol (CH_3_CH_2_CH_2_OH 99.7%), methanol (CH_3_OH 99.8%), ethanol (CH_3_CH_2_OH ≥ 99.8%), diethyl ether ((CH_3_CH_2_)_2_O ≥ 99.7%), and sodium benzene-1,3-disulfonate ((C_6_H_4_(SO_3_Na)_2_ 80%) were obtained from Sigma-Aldrich, St. Louis, MO, USA. The synthesis of NPBA and [Ag(NPBA)_2_]NO_3_ have been previously described [49]. The synthesis of **1** was carried out by following a previous reported synthesis [58].

Synthesis of [Ag(NPBA)_2_](ClO_4_) (2). A solution of NPBA (20 mg, 0.10 mmol) in 7 mL methanol was added to a separate solution containing silver perchlorate (10.7 mg, 0.05 mmol) in 3 mL of methanol. After constant stirring, a white precipitate appeared and after allowing the mixture to react under stirring for 3 h, the precipitate was filtered. A saturated solution of this complex in DMF was prepared and left for diethyl ether diffusion for 48 h in the dark, generating colorless prism-shaped crystals that were subsequently filtered and washed with diethyl ether. Yield: 26.8 mg, 87%; IR (cm^−1^): 2360, 1658, 1584, 1506, 1418, 1331, 1266, 1064, 824, 712, 622. Elemental analysis calculated for [Ag(NPBA)_2_](ClO_4_): C, 47.74; H, 3.34; N, 9.28. Found: C, 47.60; H, 3.38; N, 9.14. CCDC (Cambridge Crystallographic Data Centre): 1404799.

Synthesis of [Ag(NPBA)_2_](OTF—Triflate) (**3**). A solution of NPBA (20 mg, 0.10 mmol) in 7 mL of ethanol was added to a separate solution containing silver triflate (13.2mg, 0.05 mmol) in 3 mL of ethanol. The resulting solution was allowed to stir for 3 h; the precipitate formed was then filtered. A saturated solution of the complex in methanol was then prepared and left for diethyl ether diffusion into the reaction mixture for 72 h in the dark, generating colorless block shaped crystals. These crystals were subsequently collected, filtered, and washed with diethyl ether. Yield: 28.1 mg, 85%; IR (cm^−1^): 2367, 1682, 1604, 1520, 1424, 1337, 1266, 1033. Elemental analysis calculated for [Ag(NPBA)_2_](OTF): C, 53.78; H, 3.72; N, 10.35. Found: C, 45.96; H, 3.22; N, 8.62. CCDC: 1404800.

Synthesis of [Ag(NPBA)_2_](PF_6_) (**4**). A solution of NPBA (40 mg, 0.20 mmol) in 10 mL of propanol was added to a separate solution containing silver hexafluorophosphate (25.5 mg, 0.10 mmol) in 10 mL of propanol. Upon mixing, the mixture did not immediately produce a precipitate, even after being allowed to react for several hours. The complex could be crystallized by allowing the propanol to evaporate slowly. Yield: 53.7 mg, 82%; IR (cm^−1^): 2359, 1662, 1589, 1520, 1417, 1334, 1218, 822, 706. Elemental analysis calculated for [Ag(NPBA)_2_](PF_6_): C, 44.40; H, 3.10; N, 8.63. Found: C, 46.27; H, 3.74; N, 8.46. CCDC: 1404801.

Synthesis of [Ag(NPBA)_2_](BF_4_) (**5**). A solution of NPBA (40 mg, 0.20 mmol) in 10 mL of methanol was added to a separate solution containing silver tetrafluoroborate (19.6 mg, 0.10 mmol) in 10 mL of methanol. After allowing the mixture to react for approximately 2 h, a white precipitate appeared and was subsequently filtered. A saturated solution of this complex in DMF was prepared and left for diethyl ether diffusion over 48 h in the dark to obtain crystals, which were subsequently filtered and washed with diethyl ether. Yield: 51.3 mg, 86%; IR (cm^−1^): 1658, 1586, 1517, 1419, 1334, 1215, 1011, 822, 705, 589. Elemental analysis calculated for [Ag(NPBA)_2_](BF_4_): C, 48.77; H, 3.41; N, 9.48. Found: C, 48.84; H, 3.49; N, 9.48. CCDC: 1404802.

Synthesis of [Ag(NPBA)_2_](tosylate) (**6**). A solution of NPBA (20 mg, 0.10 mmol) in 7 mL of ethanol was added to a separate solution containing silver tosylate (14.4 mg, 0.05 mmol) in 3 mL of ethanol. After allowing the mixture to react for 3 h, a white precipitate formed and it was filtered from the reaction mixture. A saturated solution of this precipitate in methanol was left for diethyl ether diffusion for 48 h in the dark, and this generated colorless prism-shaped crystals. Yield: 28.9 mg, 84%; IR (cm^−1^): 1693, 1601, 1510, 1165, 1011, 814, 685, 561. Elemental analysis calculated for [Ag(NPBA)_2_](tosylate): C, 55.12; H, 4.03; N, 8.29. Found: C, 55.00; H, 4.16; N, 8.30. CCDC: 1532773.

Synthesis of [Ag(NPBA)_2_](Phenyl sulfonate) (**7**). A mixture of sodium benzene-1,3-disulfonate (141 mg, 0.50 mmol) and silver nitrate (170 mg, 1.0 mmol) in 2 mL of water was stirred for 5 min. Meanwhile, a solution of NPBA (190 mg, 1 mmol) was dissolved in 5 mL of methanol. After adding both solutions, there was immediate production of a white precipitate, which was dissolved by the dropwise addition of aqueous NH_3_ (2 mL). The clear solution was kept for crystallization in the dark for one week, generating silver rectangular shaped crystals. Yield 132 mg, 26.3%; IR (cm^−1^): 2358, 1674, 1591, 1504, 1415, 1327, 1292, 824, 709.

Characterization: Complexes **1**–**6** were characterized using a Nonius Kappa CCD diffractometer equipped with Mo Kα radiation (λ = 0.71073 Å). Hydrogen atoms were located and refined isotropically using SHELXL-2018. XRPD (X-ray powder diffraction) measurements were performed on a D8 diffractometer from Bruker instruments (Billerica, MA, USA) (Cu Kα radiation, *λ* = 0.154 nm) with a scan rate of 2 degrees/min. The IR spectra were recorded using a Bruker Tensor 27 FT/IR (Billerica, MA, USA) in the range of 4000–500 cm^−1^. Differential scanning calorimetry (DSC) analysis was performed in a Q2000 DSC (New Castle, DE, USA). The OM (Optical Microscopy) analysis was conducted using an Olympus IX71 optical microscope (Center Valley, PA, USA). AFM (atomic force microscopy) analysis was conducted in a Dimension FastScan AFM (Billerica, MA, USA).

## 3. Results and Discussion

Synthesis. The syntheses of the Ag complexes **1**–**7** consisted of dissolving NPBA and the corresponding silver salt into methanol or ethanol, and allowing crystallization using diethyl ether diffusion in the dark. The crystals were isolated by filtration, and they were characterized with FTIR (Fourier-transform infrared spectroscopy), XRPD (X-ray powder diffraction), and X-ray crystallography.

**Figure 1:**
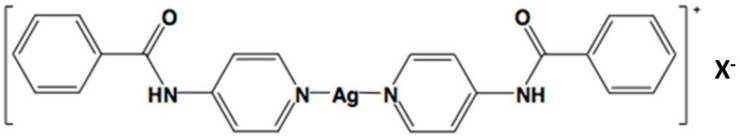
Structure of [Ag(NPBA)_2_]X, where X^−^ = NO_3_^−^ (**1**), ClO_4_^−^ (**2**), CF_3_SO_3_^−^ (**3**), PF_6_^−^ (**4**), BF_4_^−^ (**5**), CH_3_PhSO_3_^−^ (**6**), and PhSO_3_^−^ (**7**).

Structure. Silver(I) bis-*N*-(4-pyridyl) benzamide has two molecules of NPBA coordinated to Ag(I) ion in the *trans* configuration (Figure 1). The carboxamide and phenyl groups in the complex allow it to form intermolecular donor/acceptor hydrogen bonds and π–π stacking interactions, respectively. The single positive charge of the complex allowed for the study of the influence that various anions have on the arrangement of intermolecular hydrogen bonding and π–π stacking interactions in the crystalline solid state. For this study, the anions selected were NO_3_^−^ (**1**), ClO_4_^−^ (**2**), CF_3_SO_3_^−^ (**3**), PF_6_^−^ (**4**), BF_4_^−^ (**5**), CH_3_PhSO_3_^−^ (**6**), and PhSO_3_^−^ (**7**). These counterions provided a variation in the charge density and electrostatic potential, as well as varying in their abilities to hydrogen bond and engage in π–π stacking interactions.

**Figure 2:**
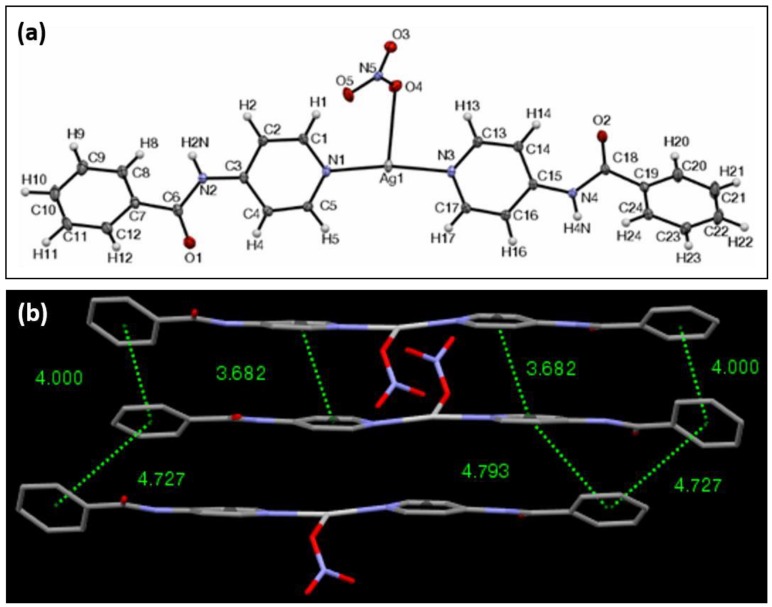
(**a**) ORTEP view of complex **1** with ellipsoids drawn at 30% probability; (**b**) View of the π–π interaction network of complex **1**, showing that the aromatic centroid-to-centroid distances are less than 4.80 Å.

The ORTEP (The Oak Ridge Thermal Ellipsoid Plot) view of Ag-complex **1** is shown in Figure 2a. It crystallizes in the triclinic crystal system in the Pī space group. The structure revealed the overall length of the ligand–Ag–ligand component, as defined by the internuclear distance from the furthest two hydrogens possible, to be 24.53 Å, and these two hydrogens formed a 166.2° angle with the silver center. The N–Ag–N bond angle is 168.6° and the planes formed by the pyridyl groups of the respective nitrogen atoms are offset by 5.0°. The two peripheral aromatic groups of the ligands form planes that intersect at an angle of 18.9°. Because the inner two pyridyl groups coordinated to the silver center are nearly coplanar, an average plane of the two can be created to be used as a reference for the outer aromatic portions of the ligand. The two outer phenyl planes of the ligands intersect this average plane at angles of 30.5° and 46.3° in the same direction. The bond lengths between the silver and the nitrogen atoms are 2.15 Å, whereas the distance between the silver and the oxygen belonging to the nitrate anion is 2.71 Å. The crystallographic values for compounds **2**–**6** are shown in Table 1.

In Ag complex **1**, the hydrogen bonding observed between the amide proton and the oxygen atom belonging to the nitrate anion are only separated by 2.11 Å. The amide oxygen also interacts with two nearby aromatic protons, with distances within 2.59 Å and 2.49 Å. The distance between the silvers on two adjacent molecules within the lattice is 3.48 Å. A remarkable feature of Ag-complex **1** is the directional π–π stacking interaction network that it exhibits (Figure 2b). The molecules stack lengthwise along the *a* crystallographic axis on top of each other, allowing for a virtually complete overlap between the corresponding aromatic elements. The average planes of adjacent complexes that participate in the π–π stacking are parallel with each other, while the peripheral phenyl planes of two adjacent π–π stacked complexes intersect at one end at an angle of 18.8° and at 18.9°. The opposing phenyl planes of two separate complexes are in parallel for one set, and for the other set, it intersects at an angle of 0.2°. There are a total of eight π-stacking interactions with centroid-to-centroid distances of 4.80 Å or less. The central complex’s aromatic centroids overlap with the centroids of the complex below it, and it is offset from the aromatic rings above it. This motif is repeated in such a way as to form a “column” of complexes where there is a slight offset in overlap at every other complex molecule. XRPD analysis of **1** revealed a consistency of the crystalline structure throughout the material (Supplementary Information, Figure S1). FTIR showed carbonyl stretching at 1740 (cm^−1^), amide stretching at 1675 (cm^−1^) and a C–N double bond at 1504 (cm^−1^).

**Figure 3:**
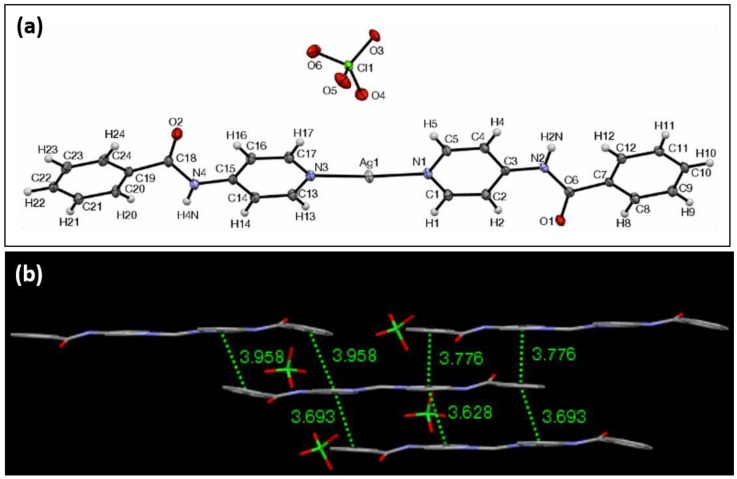
(**a**) ORTEP view of complex **2** with ellipsoids drawn at 30% probability; (**b**) The view according to the inset of the intermolecular π–π interactions of four adjacent molecules of complex **2**, highlighting the centroid–centroid distances that are less than 3.96 Å.

The ORTEP view of Ag complex **2** with ClO_4_^−^ as the counterion is shown in Figure 3a. It crystallizes in the triclinic crystal system in the Pī space group. The unit cell in this case consists of two molecules of the silver complex, each with a slightly different dimensional specification. One of the moieties measures 24.60 Å, whereas the second measures 24.63 Å in overall length, as previously defined, and they form angles of 176.5° and 179.7° with respect to the outermost aromatic protons, respectively. The N–Ag–N bond lengths of each of the silver complexes in the unit cell are 2.13 Å, 2.13 Å, and 2.12 Å, 2.12 Å for each complex. The N–Ag–N bond angles for each complex were found to be 172.3° and 176.0°. The Ag centers of the complexes were not coordinated to the perchlorate anions, as was the case with the nitrate complex. The pyridyl groups directly bonded to the Ag center are similarly not coplanar, and their respective planes intersect at an angle of 6.0° for the first moiety, and 8.8° for the second. The peripheral aromatic groups, those belonging to the phenyl rings of the ligand, intersect at an angle of 9.5° and 9.5° for each of the two complexes.

Two types of hydrogen bonds are displayed within the structure of complex **2**. One of them is between the amide proton and the oxygen of the perchlorate anion, which are separated by a distance of 2.17 Å. The other is between a solvent molecule of methanol, which was trapped within the lattice during the synthesis, and hydrogen bonds via its hydroxyl proton to the amide oxygen of the complex, with a distance of 1.98 Å. Complex **2** forms an extended π–π stacking network along three dimensions in the crystal (Figure 3b). The distance between the nearest silver ions is 5.65 Å. While the silver ion centers are farther than those found in the other anion complexes, the π–π stacking interactions are more pronounced, as one molecule of the complex can interact with as many as five other complexes. Four of the complexes are stacked in a lengthwise staggered arrangement, and they form a “sheet” with their average planes being essentially parallel, whereas the fifth complex lies slightly outside of this sheet and is diagonal to the central complex unit in a parallel displaced arrangement. Choosing any arbitrary complex molecule to analyze the π-interactions, there are seven centroid-to-centroid distances of less than 3.96 Å. An XRPD analysis of **2** revealed a consistency of crystalline structure throughout the material (Supplementary Information, Figure S2). FTIR analysis showed the carbonyl stretching at 1658 (cm^−1^).

**Figure 4:**
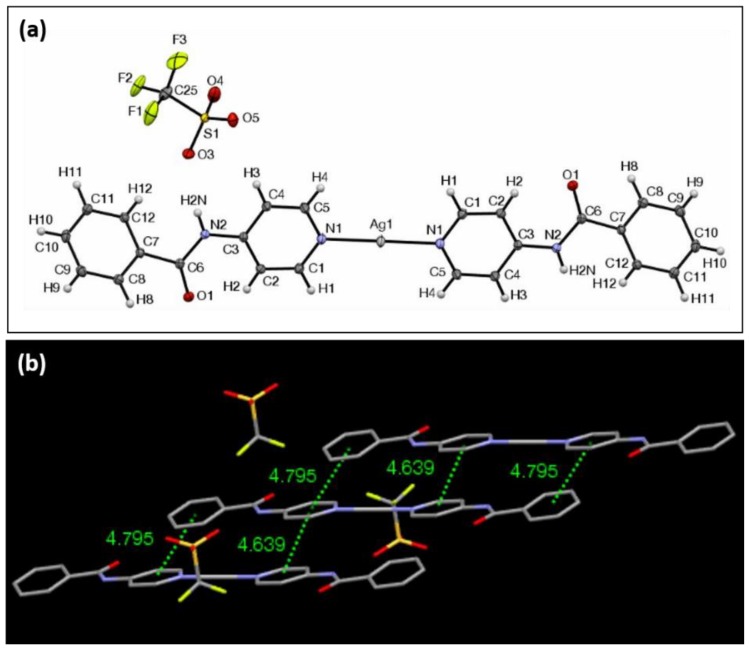
(**a**) ORTEP view of complex **3** with ellipsoids drawn at 30% probability. (**b**) The view of π–π interactions of three adjacent molecules of complex **3**, showing centroid-to-centroid distances of less than 4.80 Å.

The ORTEP view of complex **3** with CF_3_SO_3_^−^ as the counterion is shown in Figure 4a. It crystallizes in the triclinic crystal system in the Pī space group. The unit cell in this case consists of two molecules of the silver complex, as in the case of complex **2**. The first of the complexes measures at 24.63 Å, whereas the second measures at 24.48 Å in overall length with respect to the first definition that was previously given, and both form perfectly linear angles of 180.0° with respect to the outermost aromatic protons. The N–Ag–N bond lengths of each of the silver complexes in the unit cell are equivalent, unlike the previous complexes, and they measure 2.11 Å and 2.10 Å. The N–Ag–N bond angles for each were found to be equivalent for each moiety, and they are also perfectly linear at 180.0°. The Ag centers of the complexes are not coordinated to the triflouromethanesulfonate (triflate) anions. The pyridyl groups that are directly bonded to the Ag center are the closest to being coplanar in comparison with the other complexes, with their angles of intersection measuring 0.1° and 0.5°. The planes of the phenyl rings of the ligands intersect at angles of 0.8° and 0.1° for each of the two complexes. The average planes of the complexes intersect at an angle of 85.3°.

Hydrogen bonding is present within the lattice between adjacent oxygen atoms of the triflouromethanesulfonate anion and the amide proton of two adjacent complexes, and these have a distance of 2.13 Å, and 2.27 Å. The two complex molecules that participate in these interactions do not belong to the same unit cell, but their average planes also intersect at an angle of 85.3°. The closest silver-to-silver spacing is 7.47 Å. The overall structure of the lattice exhibits alternating layers of the complexes, arranged in a type of three-dimensional herringbone array (Figure 4b). Again, choosing any arbitrary complex molecule for π-interaction analysis shows that there are five π–π interactions with centroid-to-centroid distances of less than 4.80 Å. An XRPD analysis of **3** revealed that other polymorphs may be present throughout the material (Supplementary Information, Figure S3). FTIR analysis showed carbonyl stretching at 1682 (cm^−1^).

**Figure 5:**
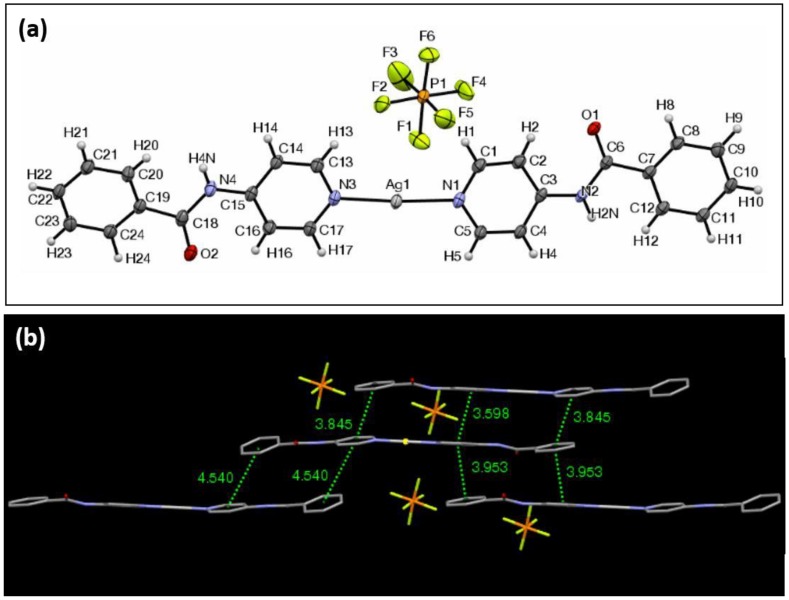
(**a**) The ORTEP view of complex **4** with ellipsoids drawn at 30% probability. (**b**) The view of the π–π interaction network of complex **4**, highlighting centroid–centroid distances of less than 4.54 Å.

The ORTEP view of complex **4** with PF_6_^−^ as the counterion is shown in Figure 5a. It crystallizes in the triclinic crystal system in the Pī space group. The overall length of the ligand–Ag–ligand component is 24.57 Å, and the two outermost hydrogens form a 175.6° angle with the silver center. The N–Ag–N bond angle is 175.8°, and the planes formed by the pyridyl groups of the respective nitrogen atoms intersect at an angle of 5.0°. The two peripheral aromatic groups of the ligands form planes that intersect at an angle of 13.6°. The two outer phenyl planes of the ligands intersect the average plane of the inner pyridyl planes at angles of 20.7° and 8.0°. The bond lengths between the silver and the nitrogen are 2.13Å and 2.14 Å. As is typical of these complexes, π–π stacking interactions predominate, as any one individual complex can form strong interactions with as many as three other complexes (Figure 5b). Unlike the case of complex **2**, the average planes of adjacent complexes that participate in the π–π stacking are not in parallel with each other. There are seven π–π interactions with centroid distances of 4.54 Å or less. XRPD analysis of **4** revealed that other polymorphs may be present throughout the material (Supplementary Information, Figure S4). FTIR analysis shows the carbonyl stretching at 1662 (cm^−1^).

**Figure 6:**
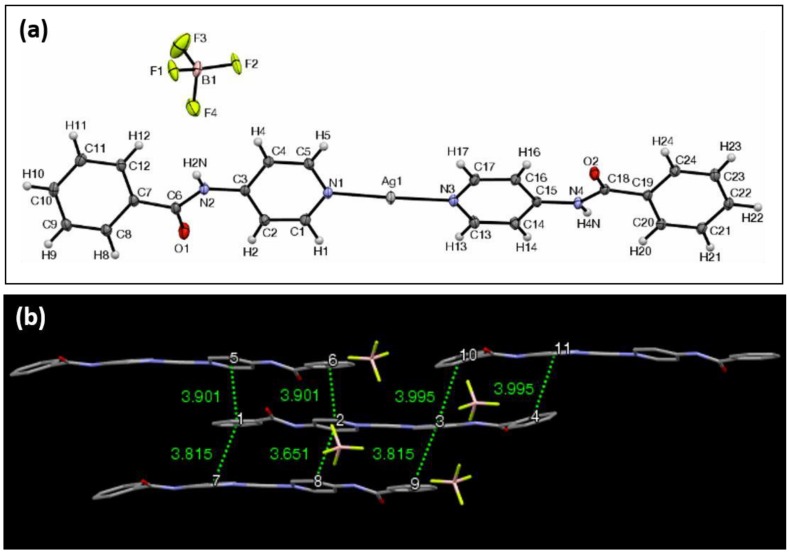
(**a**) The ORTEP view of complex **5**, with ellipsoids drawn at 30% probability. (**b**) The view of the π–π interaction network of complex **5**, highlighting the centroid-centroid distances of less than 4.00 Å.

The ORTEP view of complex **5** with BF4- as the counterion is shown in Figure 6a. It crystallizes in the triclinic crystal system in the Pī space group. The unit cell in this case consists of the anion, two molecules of the silver complex, and two molecules of methanol, the solvent used for crystallization. The two complex molecules and methanol molecules are symmetrical with respect to an inversion center between them, with the methanol molecules being disordered over their positions. The length of the complex, as previously defined in all structures, is 24.68 Å. The two N–Ag bond lengths are 2.12 and 2.12 Å. The N–Ag–N bond angle is 178.1°. The methanolic oxygen is coordinated to silver, with a distance of 2.67 Å. The pyridyl groups directly bonded to the Ag center are not coplanar, with their respective planes intersecting at an angle of 17.0°. The peripheral aromatic groups, those belonging to the phenyl rings of the ligand, intersect at an angle of 18.7°. Complex **5** forms an extended π–π stacking network along two dimensions in the crystal, and a single complex molecule forms π–π stacking interactions with three molecules in the immediate vicinity (Figure 6b). Three of those interactions are face-to-face interactions between the complexes, which helps to form a sheet-like array of complexes that are arranged in a brick-like pattern. There are seven aromatic centroid-to-centroid distances of 4.00 Å or less. XRPD analysis of **5** revealed that other polymorphs may be present throughout the material (Supplementary Information, Figure S5). The FTIR shows the carbonyl stretching at 1658 (cm^−1^).

**Figure 7:**
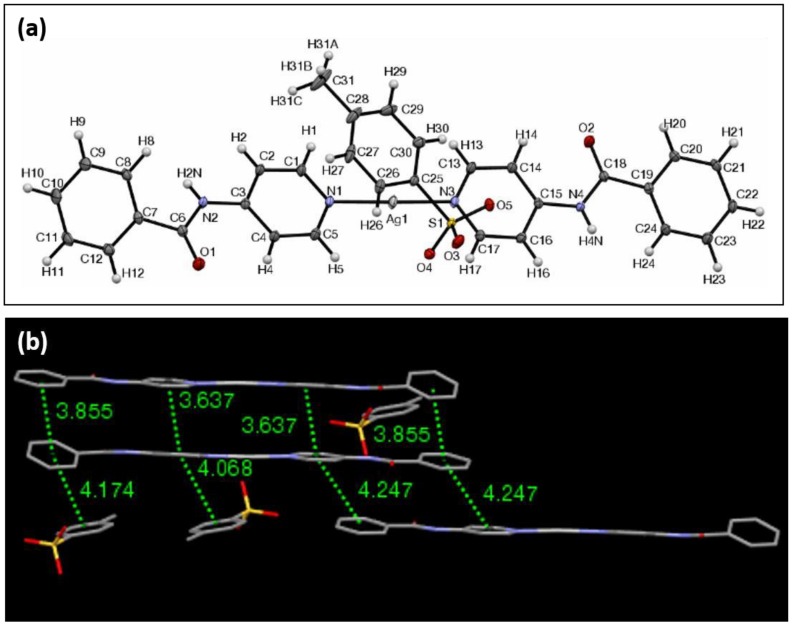
(**a**) The ORTEP view of complex **6**, with ellipsoids drawn at 30% probability. (**b**) The view according to π–π interaction network complex **6**, highlighting the centroid-to-centroid distances of 4.25 Å or less.

The ORTEP view of complex **6** with CH_3_C_6_H_4_SO_3_^−^ as the counterion is shown in Figure 7a. It crystallizes in the triclinic crystal system in the Pī space group. In this complex, the N–Ag–N bond lengths are 2.12 Å and 2.12 Å, with the overall length of the complex being 24.54 Å. The N–Ag–N angle was measured to be 176.9°, and the distance between an oxygen atom of the p-toluenesulfonate (tosylate) anion to the silver center was 2.90 Å. Once again, as typical in these complexes, the planes formed by the pyridyl groups are not coplanar, and they intersect at an angle of 6.9°. The peripheral planes formed by the exterior phenyl rings intersect at an angle of 13.9°. The angles formed by the phenyl groups and the average plane of the pyridyl groups are 26.9° and 13.1°, with both exterior groups rotating in the same direction relative to the inner plane.

Hydrogen bonds are displayed between the amide protons and the p-toluenesulfonate oxygens, whose internuclear distance is 2.01 Å. The shortest distance between any two silver centers is 3.74 Å. Complex **6** is unique in the sense that the p-toluenesulfonate anion itself contains an aromatic π system that also participates within the π–π stacking network between the ligand–Ag–ligand portions of the complex (Figure 7b). One complex molecule interacts with two other complexes and two of the p-toluenesulfonate anions. There are six π-interactions with adjacent complexes and two π-interactions with the tosylate anion, all of which are 4.25 Å or less. XRPD analysis of **6** revealed that other polymorphs may be present throughout the material (Supplementary Information, Figure S6). The FTIR, shows the carbonyl stretching at 1693 (cm^−1^).

**Figure 8:**
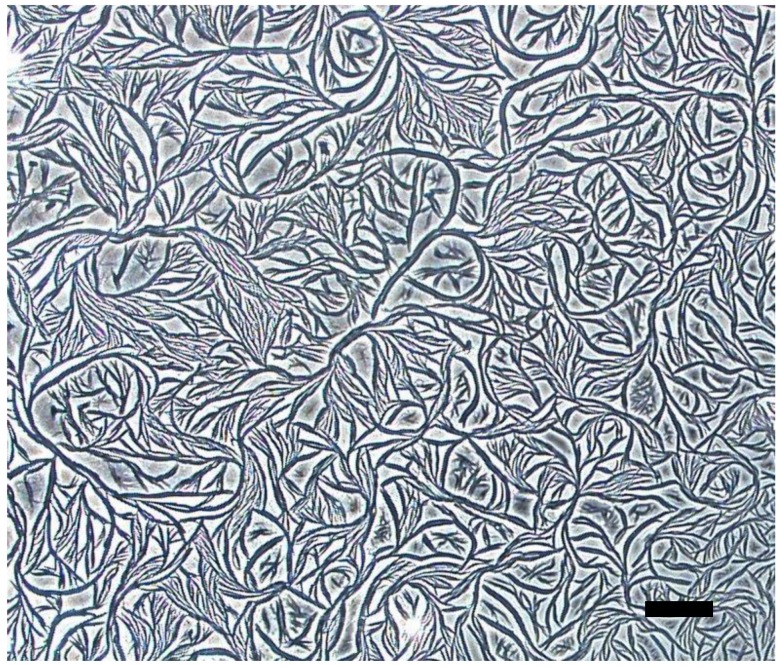
Optical microscopy images of mesomorphic phases of **6** in dimethylformamide (DMF). Scale bar is 50 μm.

Mesomorphic Phase. Ag complexes **6** and **7** formed mesomorphic phases when the DMF solutions of the complexes were heated to 130 oC and allowed to slowly evaporate (Figure 8 and Supplementary Information, Figure S7). The mesomorphic phases consist of flexible fibers, presumably of π–π stacking networks, that range in size from the nanometer to the micron scale. When the Ag complexes **1**–**5**, which do not have π–π stacking counterions, were placed under similar conditions, they formed ordinary microcrystalline materials (Supplementary Information, Figure S7). Since the Ag complexes **6** and **7** have counterions that are capable of π–π stacking via the phenyl moiety, it is likely that at high temperature in DMF, they cause bifurcation of the crystal lattice, resulting in flexible π–π-driven fibers and the mesomorphic phases. To our knowledge, this is the first example of this type of phenomena, which opens a wide range of possibilities to develop novel stacking organic materials with unique properties, and potential applications in the design of novel high-surface area organic semiconductors.

**Figure 9:**
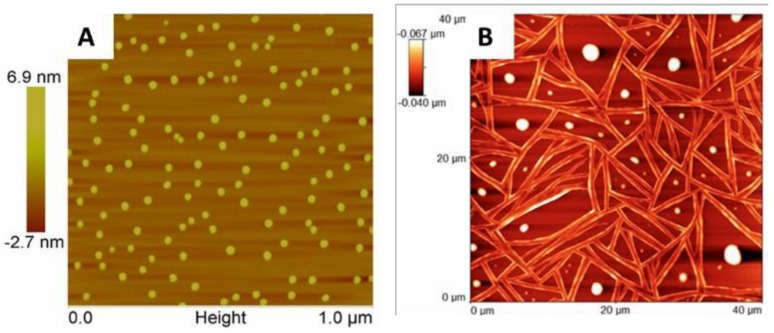
Atomic force microscopy (AFM) of crystal **6** during (**A**) nucleation and (**B**) growth in DMF at 130 °C.

Atomic Force Microscopy. Figure 9 shows the atomic force microscopy scan of the mesomorphic phase of complex **6** during its nucleation and crystal growth from DMF after heating at 130 °C. AFM indicated that the nanocrystals of complex **6** bifurcate into branches as small as 30 nm that eventually interconnect, forming long range networks that result in mesoscale phases.

**Figure 10:**
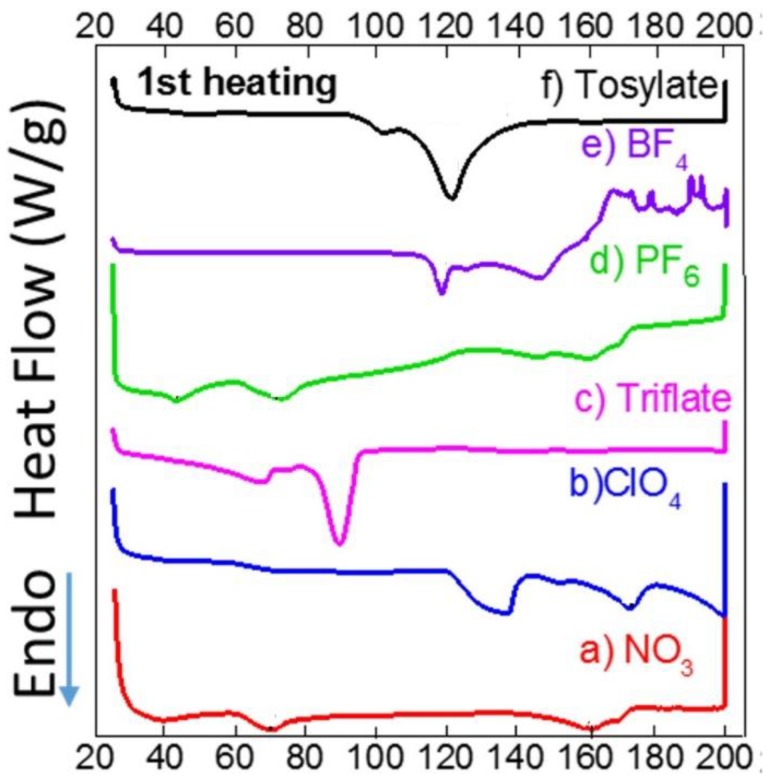
Thermographs for crystals **1**–**6** when heated from room temperature to 200 °C.

Differential Scanning Calorimetry. In order to investigate whether there were any special physical properties that contributed to the mesomorphic crystals, we studied crystals **1**–**6** with DSC. Figure 10 shows the heating thermographs of crystals **1**–**6** heated from room temperature to 200 °C. The results indicated strong endothermic points associated with melting points for crystals **2**, **3**, **4**, and **6** at 119, 76, 125, and 92 °C, respectively. Only crystal **5** appeared to decompose at over 150 °C. These results indicate that there is no remarkable thermal behavior in the Ag complexes in the solid state, and that the mesomorphic phases are most likely related to the mechanisms of crystal growth during the interference of π–π stackable counterions in solution at high temperatures.

DFT (Density Functional Theory) calculation of band gaps. Since polycyclic aromatic hydrocarbon with π–π stacking interactions in the solid-state are known to exhibit semi-conducting properties due to their low band gap properties, we carried an all-electron calculation using DFT in NRLMOL [59,60,61,62,63,64] to determine the theoretical band gaps of **1**–**6**. This was accomplished using the crystal structures of the compounds, and employing a large polarized Gaussian basis set for the calculations. The Perdew–Burke–Ernzerhof exchange-correlation functional was applied. The band gaps, as given by DFT using the pure functionals, were underestimated; therefore, the quasi particle (QP) gap was calculated using a finite model from the difference between the ionization potential and electron affinity of the finite system. The ionization energy, from the delta-SCF (Self-consistent Field) method, was well-defined within DFT, since both energies of both the neutral and charged systems were obtained as ground state energies. The QP gaps are typically larger than the optical gaps since the excitation binding energies are not taken into account; thus, they form an upper bound to the band gap of the materials under study. The molecular model consisted of clusters of two to four units of Ag complexes with the interstitial linking units. We noted that the crystal field effect was pronounced in these systems, with smaller ionization energies for the larger clusters. The necessity to consider larger clusters limited our ability to calculate the optical spectra using TDDFT (Time-dependent density functional theory). The simulated band gaps are shown in Table 2. The calculated band gap energies for complexes **1**–**6** based on their crystal structure were 3.25, 3.68, 1.48, 5.08, 1.53, and 3.55 eV, respectively. Experimental measurements of the band gaps of the Ag complexes were difficult to obtain, due to the polymorphic nature of the crystals. However, we were able to measure the experimental band gap using solid-state UV absorption spectra for the mono-morphic Ag complexes **1**–**3**, and the resulting band gaps were determined to be 3.85, 3.90, and 3.89 eV, respectively. The calculations were generally close to the experimental values in the monomorphic crystals. Interestingly, the theory predicted very low band gap energies for crystals **3** and **5**. Investigating the semi-conductive properties of the Ag complexes will be the subject of future reports. 

## 4. Conclusions

In summary, we have synthesized seven silver(I) *N*-(4-pyridyl) benzamide complexes (**1**–**7**) with varying counter ions that form a rich array of π–π stacking networks via the phenyl moiety in the ligand, which propagate in 1D, 2D and 3D crystal directions. This systematic study allowed us to discover that when the counterions are capable of π–π stacking, as is the case for **6** and **7**, they can interfere during the crystal growth in DMF at 130 °C, and induce the formation of highly branched, flexible fibers that extend from ~30 nm in diameter to the micron scale, resulting in mesomorphic phases. DFT calculations of the band gaps of the Ag complexes predicted that the Ag complexes have generally low band gaps from 3.25–5.05, some of which were closely corroborated with experimental measurements. Taken together, the ability to induce nanoscale bifurcations in π–π stacking rich materials that also exhibit low band gap properties can lead to a new generation of highly branched organic semiconductors for applications in molecular electronics.

## Figures and Tables

**Table 1:** Crystallographic data for Ag complexes **2**–**6**.

Ag Complex	2	3	4	5	6
FW (Formula Weight)	619.78	653.38	679.33	614.15	675.5
Crystal syst.	Triclinic	Triclinic	Triclinic	Triclinic	Triclinic
Space Group	Pī	Pī	Pī	Pī	Pī
a (Å)	9.8275(2)	7.46740(1)	9.9001(7)	9.8917(2)	10.7933(2)
b (Å)	12.4752(2)	10.0328(2)	10.7078(7)	10.5460(2)	10.8375(2)
c (Å)	21.0802(3)	17.8647(5)	13.0099(10)	12.3966(3)	14.0935(3)
α (degrees)	73.7175(11)	84.7974(8)	77.930(3)	74.8291(10)	67.7623(7)
β (degrees)	79.3526(10)	85.6311(8)	75.843(4)	74.8542(9)	76.8080(7)
tγ (degrees)	76.1280(7)	72.2666(16)	81.012(5)	80.3443(8)	66.1412(12)
V (Å3)	2389.18(7)	1267.89(5)	1299.65(16)	1197.89(4)	1390.03(5)
Z (Formula units)	4	2	2	2	2
D_calcd_(Mg/m^3^)	1.732	1.711	1.736	1.703	1.614
μ (mm^−1^)	1.008	0.944	0.915	0.907	0.85
Data/restrains/parameters	10,777/0/860	5640/0/435	5428/0/452	5325/0/438	6163/0/476
GOF (Goodness of fit)	1.013	1.052	1.063	1.038	1.055
Final R indices (1 > 2σ(1))	R1 = 0.0363 wR2 = 0.0843	R1 = 0.0290 wR2 = 0.0688	R1 = 0.0556 wR2 = 0.1370	R1 = 0.0334 wR2 = 0.0835	R1 = 0.0265 wR2 = 0.0634
Final R indices (all data)	R1 = 0.0565 wR2=0.0960	R1 = 0.0350 wR2 = 0.0725	R1 = 0.0744 wR2 = 0.1538	R1 = 0.0376 wR2 = 0.0865	R1 = 0.0319 wR2 = 0.0666

**Table 2:**
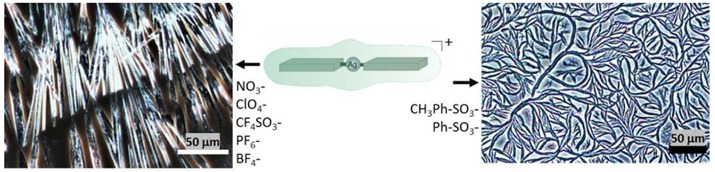
Experimental and calculated band gap energies (eV) of Ag complexes **1**–**6**.

	1	2	3	4	5	6
Experimental	3.85	3.90	3.89	-	-	-
Calculated	3.25	3.68	1.48	5.08	1.53	3.55

Mesomorphic fibers induced by π–π stacking-capable anions in silver(I) *N*-(4-pyridyl)benzamide.

## Supplementary Materials

The following are available online at http://www.mdpi.com/1996-1944/11/9/1666/s1: CCDC data of crystals **2**–**6**, XRD data, FTIR, optical microscopy, and crystallographic data are available in Supplementary Information.
