# Supplementary material for: Mesomorphic Behavior in Silver(I) N-(4-Pyridyl) Benzamide with Aromatic π–π Stacking Counterions
Source: Materials (Basel). 2018 Sep 9;11(9):1666. doi: 10.3390/ma11091666 (PMC6163786; doi:10.3390/ma11091666)
Supplement: Supplementary file 1 [file materials-11-01666-s001.pdf]

Supplementary Information

Mesomorphic behavior in silver(I) N-(4-pyridyl) benzamide with aromatic π–π stacking counterions

Isaac Torres,[a] Mauro Ruiz, [a] Hung Phan,[b] Noemi Dominguez,[a] Jacobo Garcia,[a] Thuc-Quyen Nguyen,[b] Hayden Evans,[b] Marino J. Resendiz,[c] Tunna Baruah,[d] Alejandro Metta,[a] Atta Arif,[e] Juan C. Noveron*[a]

[a]Department of Chemistry, University of Texas at El Paso, El Paso, TX 79968, USA.

[b]Department of Chemistry & Biochemistry, University of California Santa Barbara, Santa Barbara, CA 93106-9510, USA.

[c] Department of Chemistry, University of Colorado, Denver, CO 80217-3364, USA.

[d]Department of Physics, University of Texas at El Paso, El Paso, TX 79968, USA.

[e]Department of Chemistry, University of Utah, Salt Lake City, UT 84112, USA.

Correspondence: jcnoveron@utep.edu

Corresponding Author E-mail: jcnoveron@utep.edu

CCDC Data

Summary of Data CCDC 1404799 X=ClO_4^-

Compound Name:
Formula: 2(C24 H20 Ag1 N4 O2 1+),C1 H4 O1,2(Cl1 O4 1-)
Unit Cell Parameters: a 9.8275(2) b 12.4752(2) c 21.0802(3) P-1

Summary of Data CCDC 1404800 X = $CF_3SO_3^-$

Compound Name:
Formula: C24 H20 Ag1 N4 O2 1+,C1 F3 O3 S1 1-
Unit Cell Parameters: a 7.46740(10) b 10.0328(2) c 17.8647(5) P-1

Summary of Data CCDC 1404801 X = PF_6^-

Compound Name:
Formula: 2(C24 H20 Ag1 N4 O2 1+),C3 H8 O1,2(F6 P1 1-)
Unit Cell Parameters: a 9.9001(7) b 10.7078(7) c 13.0099(10) P-1

Summary of Data CCDC 1404802 X = BF_4^-

Compound Name:
Formula: C50 H42 Ag2 N8 O6 2+,2(B1 F4 1-),0.25(C2 O2)
Unit Cell Parameters: a 9.8917(2) b 10.5460(2) c 12.3966(3) P-1

Summary of Data CCDC 1532773 X = Tosyl

Compound Name:
Formula: C24 H20 Ag1 N4 O2 1+,C7 H7 O3 S1 1-
Unit Cell Parameters: a 10.7933(2) b 10.8375(2) c 14.0935(3) P-1

X-ray Powder Diffraction (XRPD):

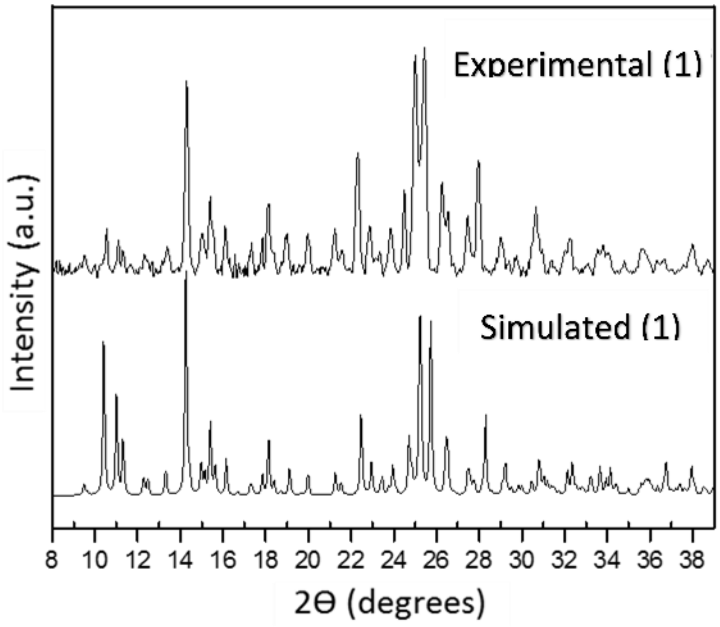

Fig. S1: Experimental and Simulated X ray diffraction patterns **1.**

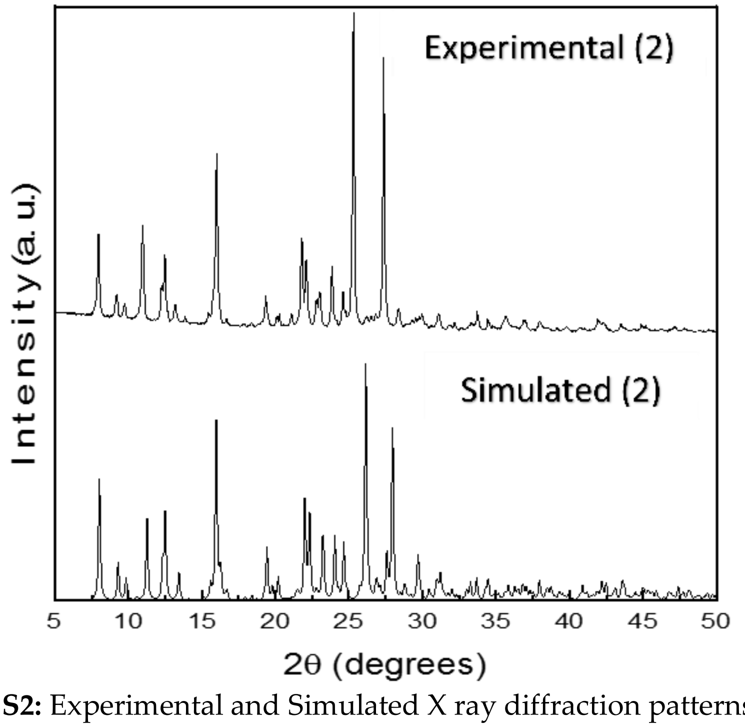

Fig. S2: Experimental and Simulated X ray diffraction patterns **2.**

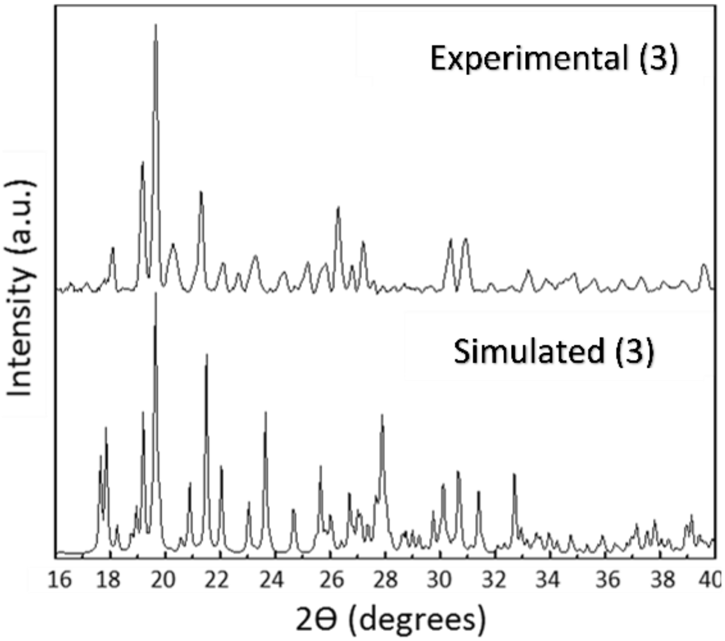

Fig. S3: Experimental and Simulated X ray diffraction patterns **3.**

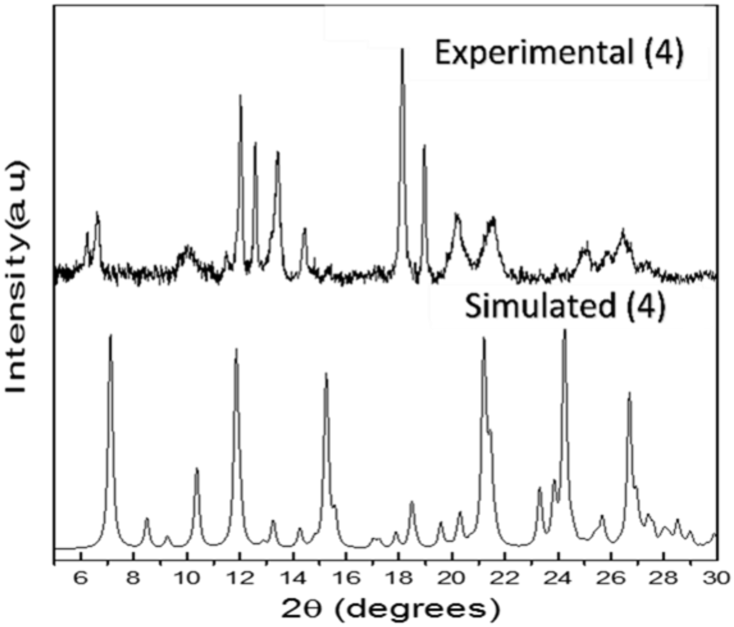

Fig. S4: Experimental and Simulated X ray diffraction patterns **4.**

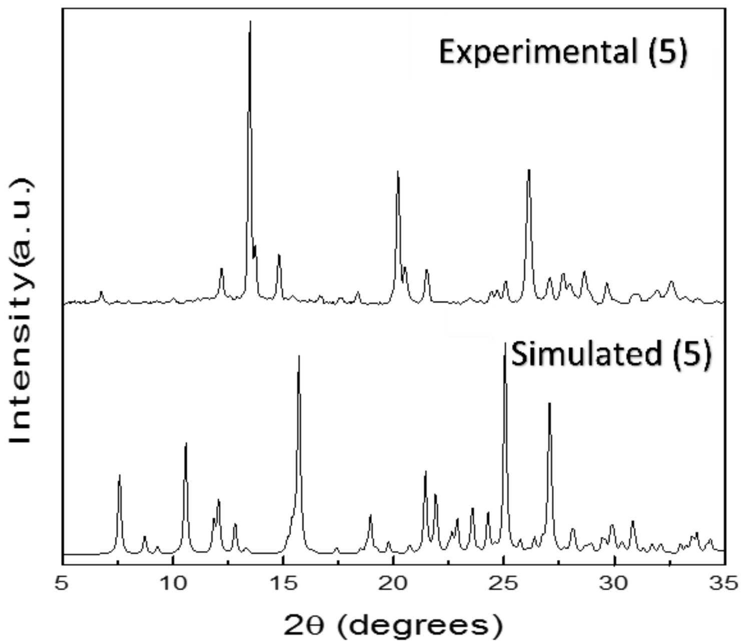

Fig. S5: Experimental and Simulated X ray diffraction patterns **5.**

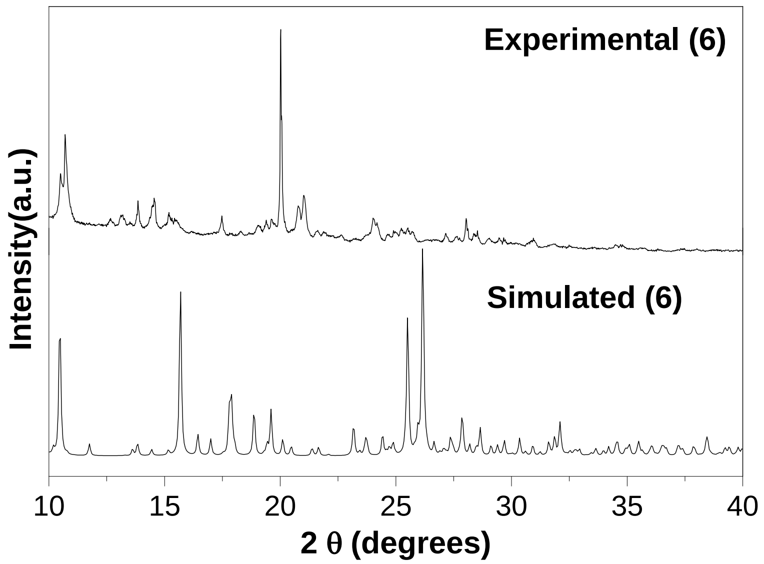

Fig. S6: Experimental and Simulated X ray diffraction patterns **6.**

Reaction Scheme:

Complex (1)

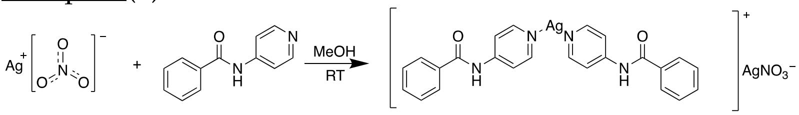

Complex (2)

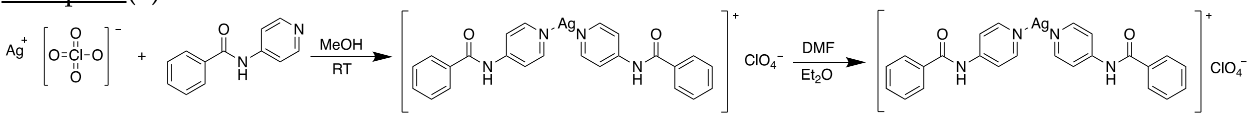

Complex (3)

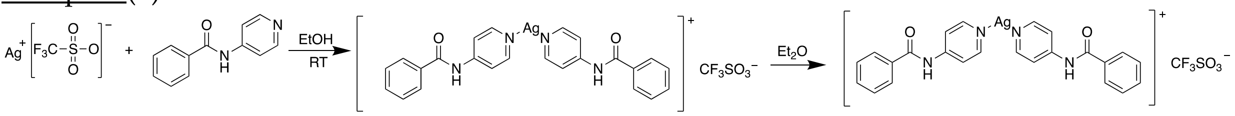

Complex (4)

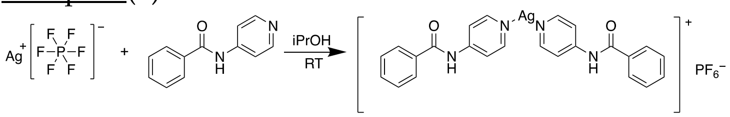

Complex (5)

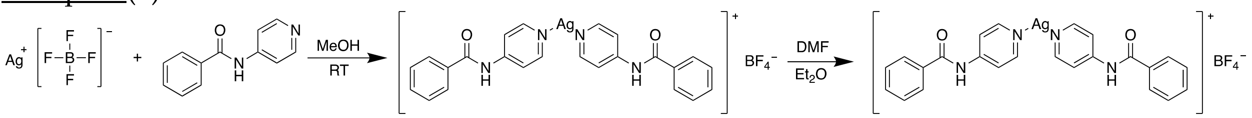

Complex (6)

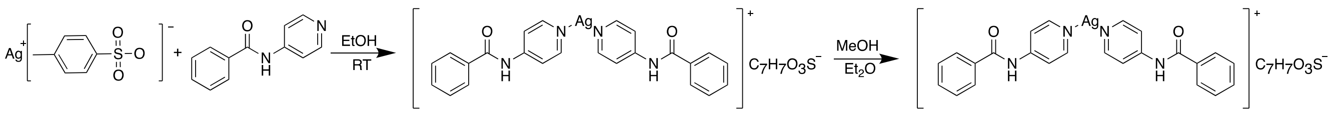

Complex (7)

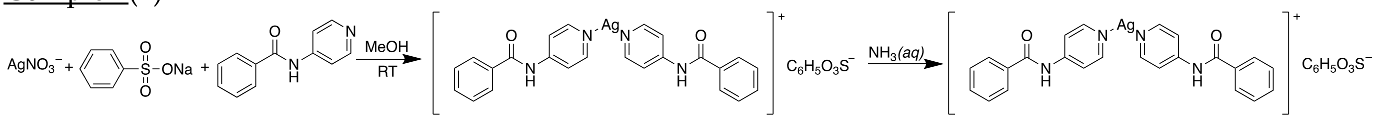

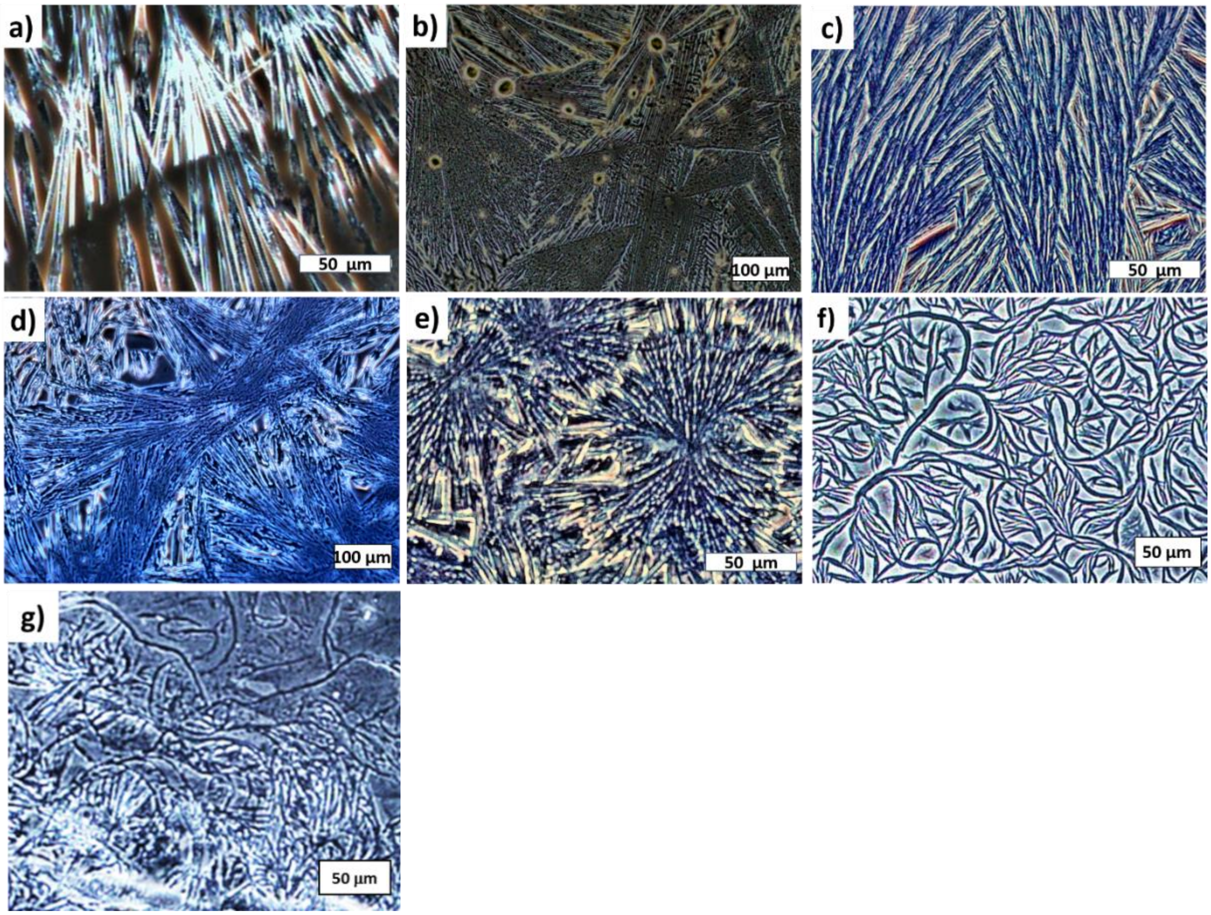

Figure S7. Optical microscopy images of crystals **1** – **7** (a – g, respectively).

Figure S8. FTIR- Spectra:

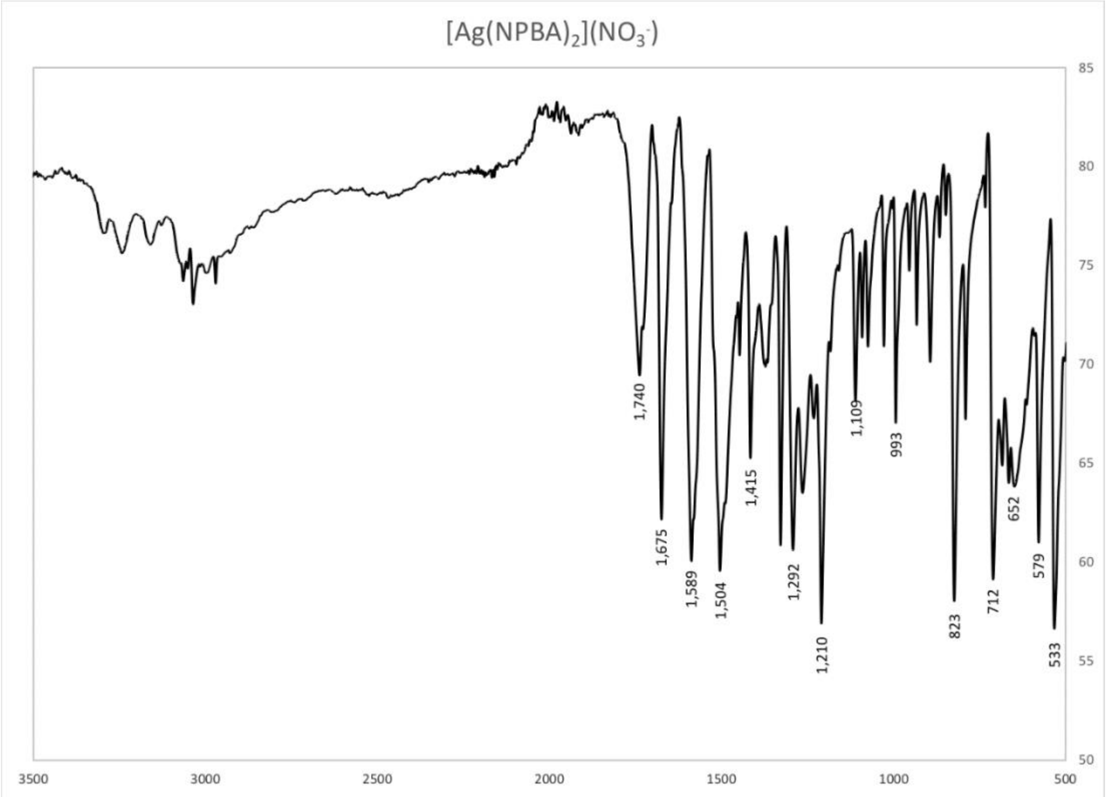

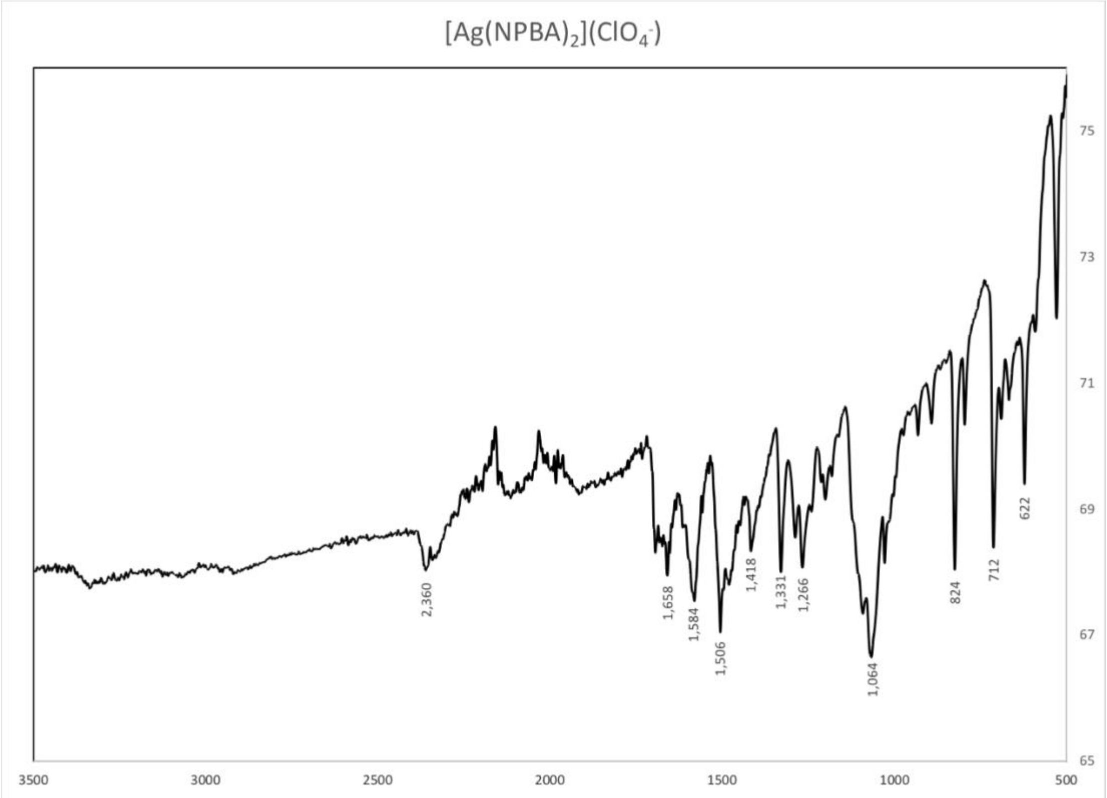

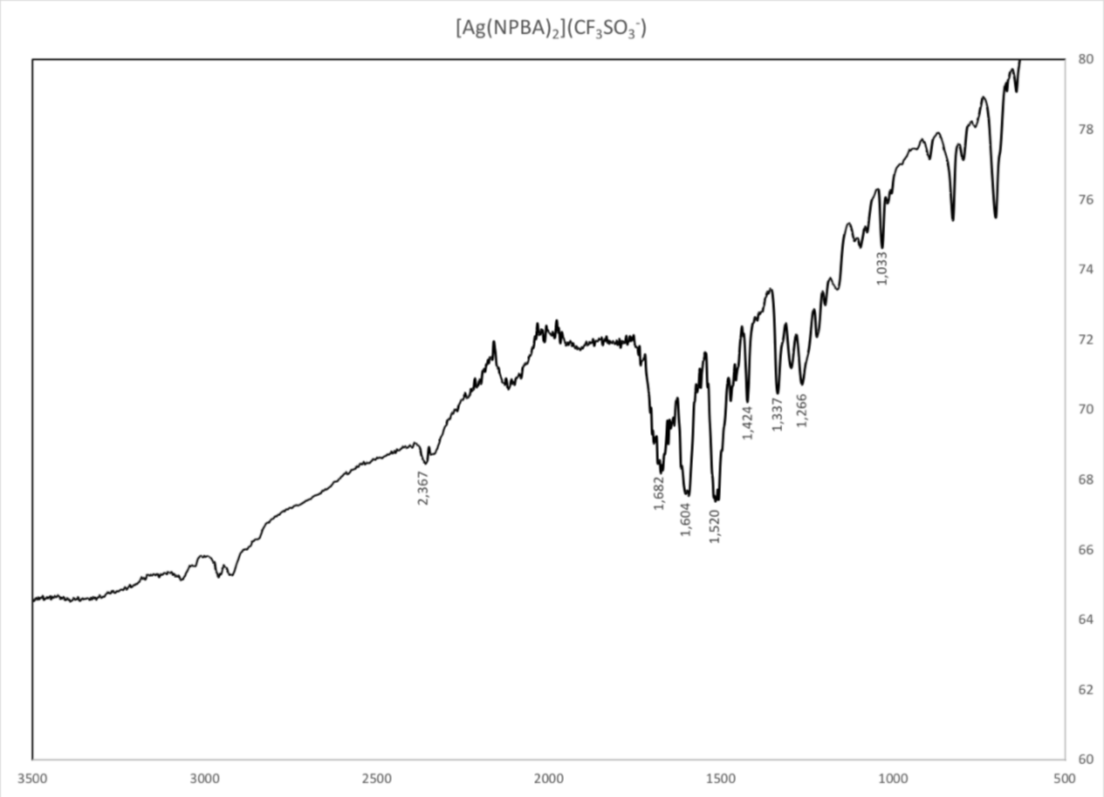
[Ag(NPBA)2](CF3SO3-)
80
78
76
74
72
70
68
66
64
62
60
3500
3000
2500
2000
1500
1000
500
2,367
1,682
1,604
1,520
1,424
1,337
1,266
1,033

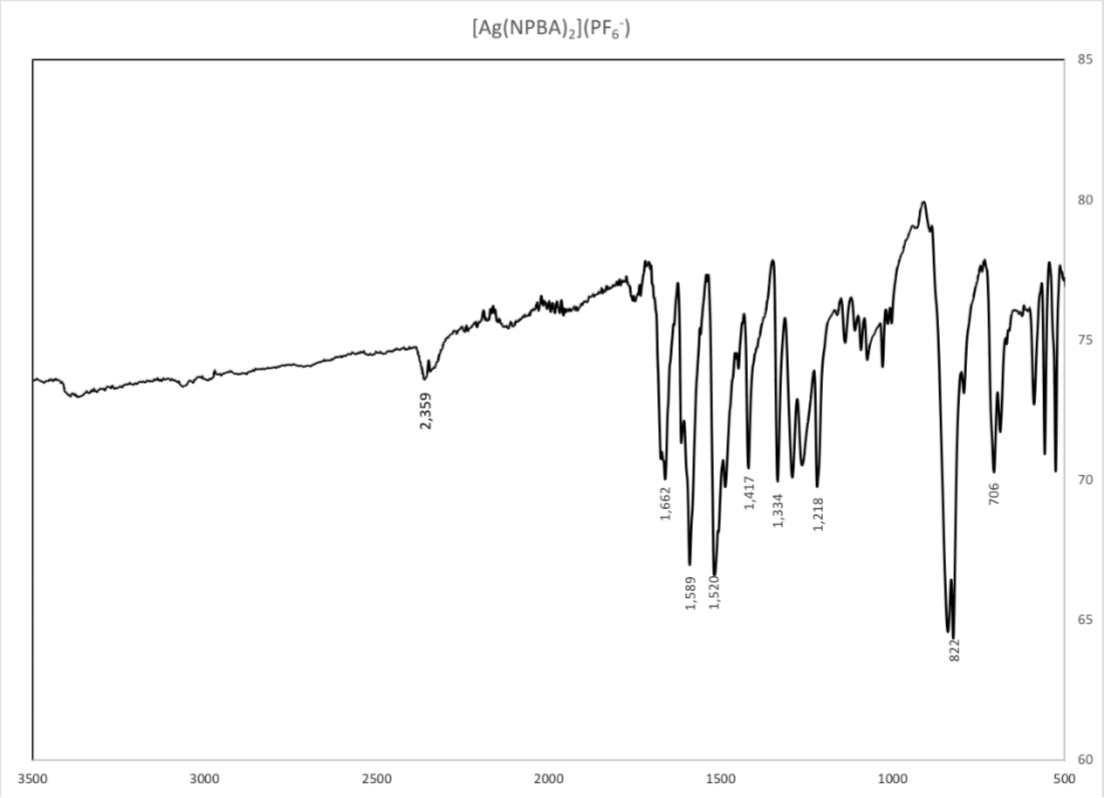
[Ag(NPBA)2](PF6-)
85
80
75
70
65
60
3500
3000
2500
2000
1500
1000
500
2,359
1,662
1,589
1,520
1,417
1,334
1,218
822
706

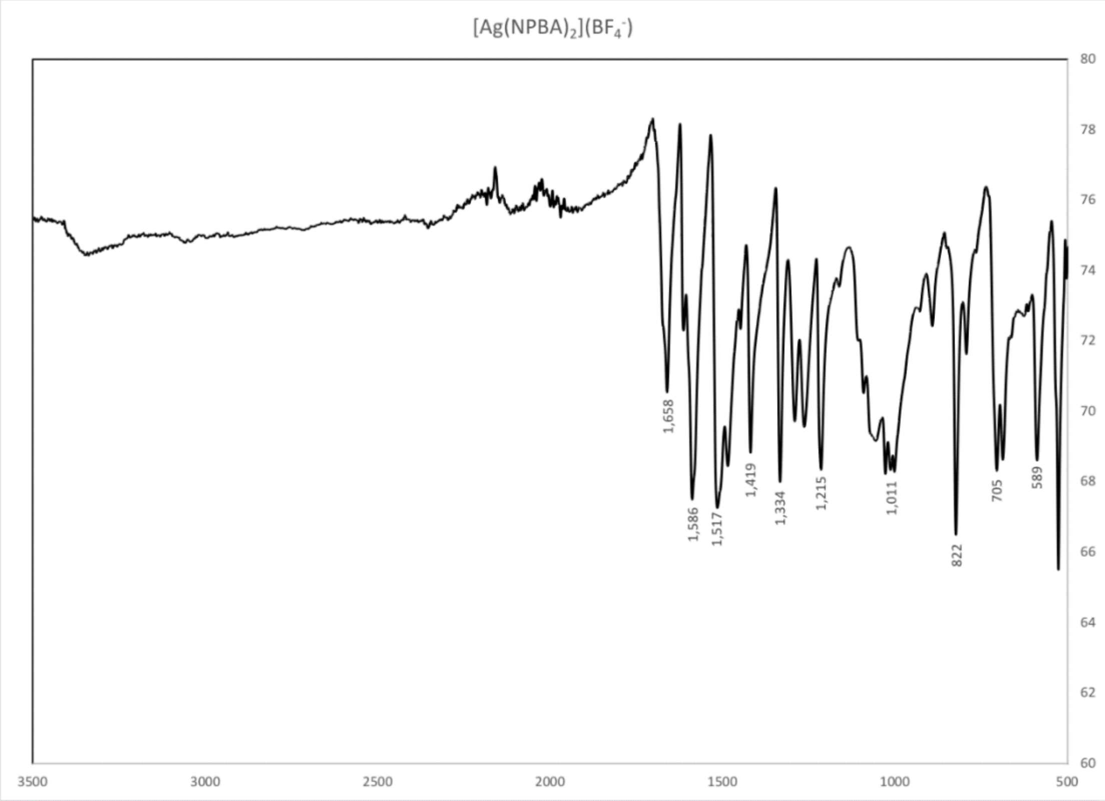

[Ag(NPBA)2](BF4-)
80
78
76
74
72
70
68
66
64
62
60
1,658
1,586
1,517
1,419
1,334
1,215
1,011
822
705
589
3500
3000
2500
2000
1500
1000
500

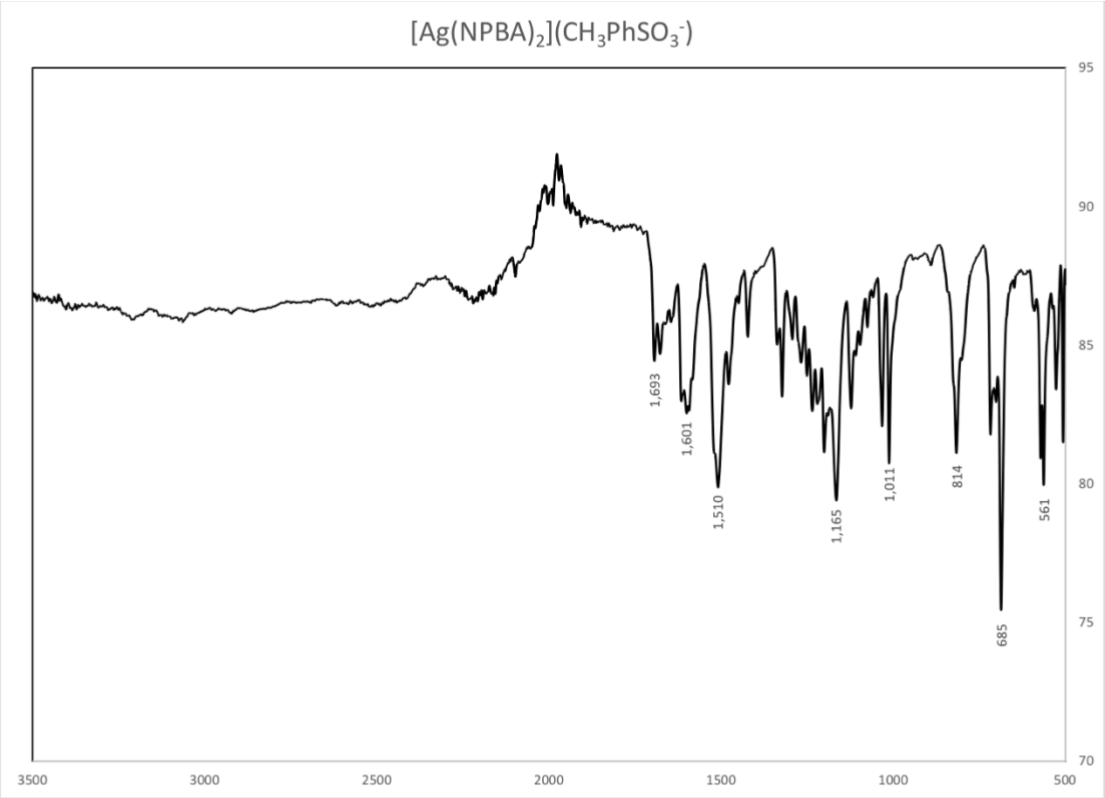

[Ag(NPBA)2](CH3PhSO3-)
95
90
85
80
75
70
1,693
1,601
1,510
1,165
1,011
814
685
561
3500
3000
2500
2000
1500
1000
500

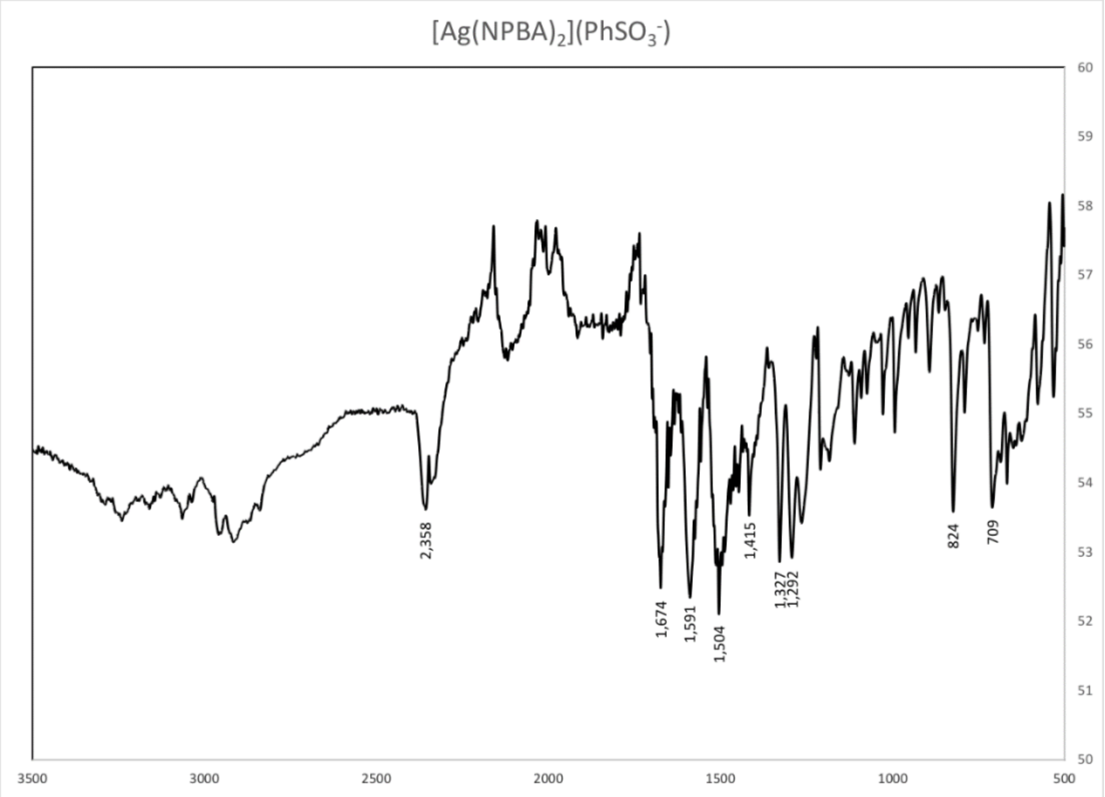

Single Crystal X-ray Diffraction Information:

Single crystals of **1 – 6** were mounted on a glass fiber with traces of viscous oil and then transferred to a Nonius Kappa CCD diffractometer equipped with Mo Kα radiation (λ = 0.71073 Å). Ten frames of data were collected at 200 K with an oscillation range of 1 deg/frame and an exposure time of 20 sec/frame. Indexing and unit cell refinement based on all observed reflection from those ten frames indicated the space groups. The reflections obtained (Θ_{max} = 27.48*) were indexed, integrated and corrected for Lorentz, polarization and absorption effects using DENZO- SMN and SCALEPAC. The structure was solved by a combination of direct methods and heavy atom using SIR 97. All of the non-hydrogen atoms were refined with anisotropic displacement coefficients. Hydrogen atoms were located and refined isotropically using SHELXL97. Scattering factors were taken from the International Tables for Crystallography, Volume C.
XRPD (X-Ray Powder Diffraction) measurements were performed on a D8 diffractometer from Bruker instruments (Cu Kα radiation, λ = 0.154 nm) with a scan rate of two degree/min. The IR spectra were recorded using a Bruker Tensor 27 FT/IR in the range of 4000-500 cm^{-1}. Differential Scanning Calorimetry (DSC) analysis was performed in a *Q2000 DSC*. For this purpose, 2.3 mg of each sample were deposited in an aluminum pan to be analyzed in a temperature range from room temperature (~ 25 °C) to 200 °C at a rate of 5 °C/min. Optical microscopy (OM) was used to observe the morphology of NPBA with different ligands. The OM analysis was conducted on an Olympus IX71 optical microscope. The preparation of samples for OM was accomplished by dissolving 5 mg of each sample in 1 ml of Dimethylformamide (DMF). Next, some portion of each dissolution was deposited on a microscope slide and then heated at 130 °C for 5 min. AFM (atomic force microscopy) analysis was conducted in a Dimension FastScan AFM. To prepare the sample certain amount of [Ag(NPBA)2](Tosylate) (6) was dissolved in DMF. Then, a drop of this solution was placed on top of a mica substrate and annealed at 130 °C for 5 minutes before analysis.

Tables for selected bond angles and distances.

Table S1: Bond lengths [Å] and angles [°] for (**2**).

Ag(1)-N(3)	2.121(2)
Ag(1)-N(1)	2.122(2)
Ag(2)-N(1A)	2.131(2)
Ag(2)-N(3A)	2.133(2)
Cl(1)-O(6')	1.276(17)
Cl(1)-O(5')	1.376(18)
Cl(1)-O(4)	1.399(2)
Cl(1)-O(6)	1.443(3)
Cl(1)-O(3)	1.4468(17)
Cl(1)-O(5)	1.448(3)
Cl(1)-O(4')	1.574(14)
Cl(2)-O(8')	1.306(12)
Cl(2)-O(9)	1.383(3)
Cl(2)-O(10)	1.402(3)
Cl(2)-O(10')	1.417(13)
Cl(2)-O(7)	1.4337(18)
Cl(2)-O(8)	1.462(3)
Cl(2)-O(9')	1.565(10)
O(1)-C(6)	1.228(3)
O(1A)-C(6A)	1.228(3)
O(2)-C(18)	1.228(3)
O(2A)-C(18A)	1.233(3)
N(1)-C(1)	1.342(3)
N(1)-C(5)	1.353(3)
N(1A)-C(1A)	1.350(3)
N(1A)-C(5A)	1.352(3)
N(2)-C(6)	1.372(3)
N(2)-C(3)	1.395(3)
N(2)-H(2N)	0.88(3)
N(2A)-C(6A)	1.367(4)
N(2A)-C(3A)	1.397(3)

N(2A)-H(2NA)	0.78(3)
N(3)-C(17)	1.342(3)
N(3)-C(13)	1.351(3)
N(3A)-C(13A)	1.345(3)
N(3A)-C(17A)	1.349(3)
N(4)-C(18)	1.364(4)
N(4)-C(15)	1.400(3)
N(4)-H(4N)	0.82(3)
N(4A)-C(18A)	1.366(3)
N(4A)-C(15A)	1.398(3)
N(4A)-H(4NA)	0.86(3)
C(1)-C(2)	1.375(4)
C(1)-H(1)	1.00(3)
C(1A)-C(2A)	1.374(4)
C(1A)-H(1A)	1.01(3)
C(2)-C(3)	1.393(3)
C(2)-H(2)	0.89(3)
C(2A)-C(3A)	1.389(4)
C(2A)-H(2A)	0.89(3)
C(3)-C(4)	1.399(4)
C(3A)-C(4A)	1.395(4)
C(4)-C(5)	1.375(4)
C(4)-H(4)	0.95(3)
C(4A)-C(5A)	1.372(4)
C(4A)-H(5A)	0.92(3)
C(5)-H(5)	0.89(3)
C(5A)-H(4A)	0.97(3)
C(6)-C(7)	1.494(3)
C(6A)-C(7A)	1.497(3)
C(7)-C(8)	1.389(4)
C(7)-C(12)	1.397(3)
C(7A)-C(12A)	1.393(4)
C(7A)-C(8A)	1.400(4)
C(8)-C(9)	1.390(4)
C(8)-H(8)	0.97(3)
C(8A)-C(9A)	1.379(4)

C(8A)-H(8A)	1.07(3)
C(9)-C(10)	1.392(4)
C(9)-H(9)	0.92(3)
C(9A)-C(10A)	1.385(4)
C(9A)-H(9A)	0.93(3)
C(10)-C(11)	1.383(4)
C(10)-H(10)	0.93(3)
C(10A)-C(11A)	1.389(4)
C(10A)-H(10A)	0.95(3)
C(11)-C(12)	1.392(3)
C(11)-H(11)	0.96(3)
C(11A)-C(12A)	1.387(4)
C(11A)-H(11A)	0.98(4)
C(12)-H(12)	0.91(3)
C(12A)-H(12A)	0.88(3)
C(13)-C(14)	1.370(4)
C(13)-H(13)	0.87(3)
C(13A)-C(14A)	1.375(4)
C(13A)-H(13A)	0.87(3)
C(14)-C(15)	1.394(4)
C(14)-H(14)	0.96(3)
C(14A)-C(15A)	1.399(4)
C(14A)-H(14A)	0.92(3)
C(15)-C(16)	1.395(3)
C(15A)-C(16A)	1.391(3)
C(16)-C(17)	1.375(4)
C(16)-H(16)	0.91(3)
C(16A)-C(17A)	1.375(4)
C(16A)-H(16A)	0.86(3)
C(17)-H(17)	1.01(3)
C(17A)-H(17A)	1.03(3)
C(18)-C(19)	1.495(3)
C(18A)-C(19A)	1.496(3)
C(19)-C(20)	1.392(3)
C(19)-C(24)	1.395(4)
C(19A)-C(24A)	1.387(4)

C(19A)-C(20A)	1.405(3)
C(20)-C(21)	1.388(4)
C(20)-H(20)	0.86(3)
C(20A)-C(21A)	1.382(3)
C(20A)-H(20A)	0.95(3)
C(21)-C(22)	1.383(4)
C(21)-H(21)	0.93(3)
C(21A)-C(22A)	1.387(4)
C(21A)-H(21A)	0.98(3)
C(22)-C(23)	1.391(4)
C(22)-H(22)	0.95(3)
C(22A)-C(23A)	1.389(3)
C(22A)-H(22A)	0.89(3)
C(23)-C(24)	1.380(4)
C(23)-H(23)	0.98(3)
C(23A)-C(24A)	1.384(3)
C(23A)-H(23A)	0.94(3)
C(24)-H(24)	1.01(3)
C(24A)-H(24A)	0.99(3)
C(25)-O(11)	1.461(5)
C(25)-H(25A)	0.9800
C(25)-H(25B)	0.9800
C(25)-H(25C)	0.9800
O(11)-H(11B)	0.8400

N(3)-Ag(1)-N(1)	175.96(8)
N(1A)-Ag(2)-N(3A)	172.33(8)
O(6')-Cl(1)-O(5')	122.8(10)
O(6')-Cl(1)-O(4)	119.4(7)
O(5')-Cl(1)-O(4)	73.3(8)
O(6')-Cl(1)-O(6)	24.2(6)
O(5')-Cl(1)-O(6)	145.6(8)
O(4)-Cl(1)-O(6)	111.1(2)
O(6')-Cl(1)-O(3)	119.2(7)
O(5')-Cl(1)-O(3)	100.5(7)
O(4)-Cl(1)-O(3)	112.24(14)

O(6)-Cl(1)-O(3)	108.24(17)
O(6')-Cl(1)-O(5)	84.2(6)
O(5')-Cl(1)-O(5)	42.6(9)
O(4)-Cl(1)-O(5)	107.9(2)
O(6)-Cl(1)-O(5)	108.4(2)
O(3)-Cl(1)-O(5)	108.83(13)
O(6')-Cl(1)-O(4')	105.6(8)
O(5')-Cl(1)-O(4')	102.3(10)
O(4)-Cl(1)-O(4')	29.2(5)
O(6)-Cl(1)-O(4')	88.9(6)
O(3)-Cl(1)-O(4')	103.9(5)
O(5)-Cl(1)-O(4')	135.1(6)
O(8')-Cl(2)-O(9)	74.8(6)
O(8')-Cl(2)-O(10)	121.0(5)
O(9)-Cl(2)-O(10)	114.8(3)
O(8')-Cl(2)-O(10')	117.5(7)
O(9)-Cl(2)-O(10')	139.7(5)
O(10)-Cl(2)-O(10')	24.9(4)
O(8')-Cl(2)-O(7)	120.6(5)
O(9)-Cl(2)-O(7)	106.2(2)
O(10)-Cl(2)-O(7)	112.10(19)
O(10')-Cl(2)-O(7)	99.4(5)
O(8')-Cl(2)-O(8)	31.8(5)
O(9)-Cl(2)-O(8)	106.5(3)
O(10)-Cl(2)-O(8)	108.39(17)
O(10')-Cl(2)-O(8)	93.9(5)
O(7)-Cl(2)-O(8)	108.56(16)
O(8')-Cl(2)-O(9')	101.6(6)
O(9)-Cl(2)-O(9')	35.3(4)
O(10)-Cl(2)-O(9')	81.5(4)
O(10')-Cl(2)-O(9')	106.2(6)
O(7)-Cl(2)-O(9')	111.1(4)
O(8)-Cl(2)-O(9')	131.3(4)
C(1)-N(1)-C(5)	116.6(2)
C(1)-N(1)-Ag(1)	119.01(15)
C(5)-N(1)-Ag(1)	124.34(18)

C(1A)-N(1A)-C(5A)	116.6(2)
C(1A)-N(1A)-Ag(2)	121.89(17)
C(5A)-N(1A)-Ag(2)	121.38(17)
C(6)-N(2)-C(3)	127.1(2)
C(6)-N(2)-H(2N)	120(2)
C(3)-N(2)-H(2N)	113(2)
C(6A)-N(2A)-C(3A)	127.0(2)
C(6A)-N(2A)-H(2NA)	121(2)
C(3A)-N(2A)-H(2NA)	112(2)
C(17)-N(3)-C(13)	116.8(2)
C(17)-N(3)-Ag(1)	121.98(16)
C(13)-N(3)-Ag(1)	121.10(18)
C(13A)-N(3A)-C(17A)	116.4(2)
C(13A)-N(3A)-Ag(2)	123.13(18)
C(17A)-N(3A)-Ag(2)	120.45(16)
C(18)-N(4)-C(15)	126.0(2)
C(18)-N(4)-H(4N)	117(2)
C(15)-N(4)-H(4N)	117(2)
C(18A)-N(4A)-C(15A)	127.2(2)
C(18A)-N(4A)-H(4NA)	120(2)
C(15A)-N(4A)-H(4NA)	113(2)
N(1)-C(1)-C(2)	124.0(2)
N(1)-C(1)-H(1)	117.6(19)
C(2)-C(1)-H(1)	118.4(19)
N(1A)-C(1A)-C(2A)	124.1(3)
N(1A)-C(1A)-H(1A)	119.5(18)
C(2A)-C(1A)-H(1A)	116.4(18)
C(1)-C(2)-C(3)	119.2(3)
C(1)-C(2)-H(2)	119.1(17)
C(3)-C(2)-H(2)	121.7(17)
C(1A)-C(2A)-C(3A)	118.9(3)
C(1A)-C(2A)-H(2A)	118.9(19)
C(3A)-C(2A)-H(2A)	122.3(19)
C(2)-C(3)-N(2)	124.1(2)
C(2)-C(3)-C(4)	117.5(2)
N(2)-C(3)-C(4)	118.4(2)

C(2A)-C(3A)-C(4A)	117.7(2)
C(2A)-C(3A)-N(2A)	124.1(2)
C(4A)-C(3A)-N(2A)	118.2(2)
C(5)-C(4)-C(3)	119.5(2)
C(5)-C(4)-H(4)	121.3(18)
C(3)-C(4)-H(4)	119.2(18)
C(5A)-C(4A)-C(3A)	120.0(2)
C(5A)-C(4A)-H(5A)	117.4(19)
C(3A)-C(4A)-H(5A)	122.6(19)
N(1)-C(5)-C(4)	123.2(2)
N(1)-C(5)-H(5)	118.9(18)
C(4)-C(5)-H(5)	117.9(18)
N(1A)-C(5A)-C(4A)	122.8(2)
N(1A)-C(5A)-H(4A)	116.9(18)
C(4A)-C(5A)-H(4A)	120.3(18)
O(1)-C(6)-N(2)	122.9(2)
O(1)-C(6)-C(7)	121.3(2)
N(2)-C(6)-C(7)	115.72(19)
O(1A)-C(6A)-N(2A)	122.4(2)
O(1A)-C(6A)-C(7A)	121.0(2)
N(2A)-C(6A)-C(7A)	116.6(2)
C(8)-C(7)-C(12)	119.2(2)
C(8)-C(7)-C(6)	117.3(2)
C(12)-C(7)-C(6)	123.5(2)
C(12A)-C(7A)-C(8A)	119.1(2)
C(12A)-C(7A)-C(6A)	123.5(2)
C(8A)-C(7A)-C(6A)	117.4(2)
C(7)-C(8)-C(9)	120.5(2)
C(7)-C(8)-H(8)	115.6(17)
C(9)-C(8)-H(8)	123.7(16)
C(9A)-C(8A)-C(7A)	120.6(2)
C(9A)-C(8A)-H(8A)	123.8(17)
C(7A)-C(8A)-H(8A)	115.6(17)
C(8)-C(9)-C(10)	120.1(2)
C(8)-C(9)-H(9)	119.4(17)
C(10)-C(9)-H(9)	120.5(17)

C(8A)-C(9A)-C(10A)	120.2(2)
C(8A)-C(9A)-H(9A)	119.6(16)
C(10A)-C(9A)-H(9A)	120.1(16)
C(11)-C(10)-C(9)	119.7(2)
C(11)-C(10)-H(10)	118.6(17)
C(9)-C(10)-H(10)	121.7(17)
C(9A)-C(10A)-C(11A)	119.6(2)
C(9A)-C(10A)-H(10A)	120.9(16)
C(11A)-C(10A)-H(10A)	119.4(16)
C(10)-C(11)-C(12)	120.4(2)
C(10)-C(11)-H(11)	124.5(18)
C(12)-C(11)-H(11)	115.2(18)
C(12A)-C(11A)-C(10A)	120.6(2)
C(12A)-C(11A)-H(11A)	117(2)
C(10A)-C(11A)-H(11A)	123(2)
C(11)-C(12)-C(7)	120.1(2)
C(11)-C(12)-H(12)	118.1(16)
C(7)-C(12)-H(12)	121.7(16)
C(11A)-C(12A)-C(7A)	119.9(2)
C(11A)-C(12A)-H(12A)	120(2)
C(7A)-C(12A)-H(12A)	120(2)
N(3)-C(13)-C(14)	123.1(2)
N(3)-C(13)-H(13)	119.9(19)
C(14)-C(13)-H(13)	116.9(19)
N(3A)-C(13A)-C(14A)	124.0(2)
N(3A)-C(13A)-H(13A)	118.7(17)
C(14A)-C(13A)-H(13A)	117.3(17)
C(13)-C(14)-C(15)	119.6(2)
C(13)-C(14)-H(14)	117.5(19)
C(15)-C(14)-H(14)	122.9(19)
C(13A)-C(14A)-C(15A)	119.0(2)
C(13A)-C(14A)-H(14A)	123.2(18)
C(15A)-C(14A)-H(14A)	117.6(18)
C(14)-C(15)-C(16)	117.9(2)
C(14)-C(15)-N(4)	118.3(2)
C(16)-C(15)-N(4)	123.8(2)

C(16A)-C(15A)-N(4A)	124.1(2)
C(16A)-C(15A)-C(14A)	117.5(2)
N(4A)-C(15A)-C(14A)	118.4(2)
C(17)-C(16)-C(15)	118.6(2)
C(17)-C(16)-H(16)	122.0(18)
C(15)-C(16)-H(16)	119.3(18)
C(17A)-C(16A)-C(15A)	119.5(3)
C(17A)-C(16A)-H(16A)	119.0(19)
C(15A)-C(16A)-H(16A)	121.4(19)
N(3)-C(17)-C(16)	124.0(2)
N(3)-C(17)-H(17)	122.1(17)
C(16)-C(17)-H(17)	113.8(17)
N(3A)-C(17A)-C(16A)	123.6(2)
N(3A)-C(17A)-H(17A)	116.4(17)
C(16A)-C(17A)-H(17A)	119.9(17)
O(2)-C(18)-N(4)	122.3(2)
O(2)-C(18)-C(19)	120.8(2)
N(4)-C(18)-C(19)	116.9(2)
O(2A)-C(18A)-N(4A)	122.9(2)
O(2A)-C(18A)-C(19A)	120.6(2)
N(4A)-C(18A)-C(19A)	116.48(19)
C(20)-C(19)-C(24)	119.5(2)
C(20)-C(19)-C(18)	123.2(2)
C(24)-C(19)-C(18)	117.2(2)
C(24A)-C(19A)-C(20A)	119.3(2)
C(24A)-C(19A)-C(18A)	117.8(2)
C(20A)-C(19A)-C(18A)	123.0(2)
C(21)-C(20)-C(19)	119.6(2)
C(21)-C(20)-H(20)	117.9(19)
C(19)-C(20)-H(20)	122.5(19)
C(21A)-C(20A)-C(19A)	119.9(2)
C(21A)-C(20A)-H(20A)	118.1(17)
C(19A)-C(20A)-H(20A)	121.9(17)
C(22)-C(21)-C(20)	120.7(2)
C(22)-C(21)-H(21)	121(2)
C(20)-C(21)-H(21)	118(2)

C(20A)-C(21A)-C(22A)	120.5(2)
C(20A)-C(21A)-H(21A)	116.2(18)
C(22A)-C(21A)-H(21A)	123.2(18)
C(21)-C(22)-C(23)	119.7(2)
C(21)-C(22)-H(22)	120.1(15)
C(23)-C(22)-H(22)	120.2(15)
C(21A)-C(22A)-C(23A)	119.5(2)
C(21A)-C(22A)-H(22A)	119(2)
C(23A)-C(22A)-H(22A)	121(2)
C(24)-C(23)-C(22)	119.9(2)
C(24)-C(23)-H(23)	121.2(16)
C(22)-C(23)-H(23)	118.7(17)
C(24A)-C(23A)-C(22A)	120.4(2)
C(24A)-C(23A)-H(23A)	120.9(17)
C(22A)-C(23A)-H(23A)	118.7(17)
C(23)-C(24)-C(19)	120.5(2)
C(23)-C(24)-H(24)	124.5(18)
C(19)-C(24)-H(24)	115.0(18)
C(23A)-C(24A)-C(19A)	120.3(2)
C(23A)-C(24A)-H(24A)	123.5(16)
C(19A)-C(24A)-H(24A)	116.2(16)

Table S2: Bond lengths [Å] and angles [°] for (**3**).

Ag(1)-N(1)#1	2.0961(15)
Ag(1)-N(1)	2.0961(15)
Ag(2)-N(3)#2	2.1052(19)
Ag(2)-N(3)	2.1052(19)
S(1)-O(4)	1.4237(16)
S(1)-O(3)	1.4304(15)
S(1)-O(5)	1.4403(14)
S(1)-C(25)	1.815(2)
F(1)-C(25)	1.320(3)
F(2)-C(25)	1.315(3)
F(3)-C(25)	1.320(3)

O(1)-C(6)	1.220(2)
O(2)-C(18)	1.221(3)
N(1)-C(1)	1.340(3)
N(1)-C(5)	1.346(3)
N(2)-C(6)	1.376(2)
N(2)-C(3)	1.397(2)
N(2)-H(2N)	0.84(2)
N(3)-C(17)	1.344(3)
N(3)-C(13)	1.347(3)
N(4)-C(18)	1.375(3)
N(4)-C(15)	1.392(3)
N(4)-H(4N)	0.83(3)
C(1)-C(2)	1.381(3)
C(1)-H(1)	0.96(2)
C(2)-C(3)	1.392(3)
C(2)-H(2)	0.89(2)
C(3)-C(4)	1.394(3)
C(4)-C(5)	1.373(3)
C(4)-H(3)	0.92(3)
C(5)-H(4)	0.93(3)
C(6)-C(7)	1.499(3)
C(7)-C(12)	1.395(3)
C(7)-C(8)	1.396(3)
C(8)-C(9)	1.387(3)
C(8)-H(8)	0.98(2)
C(9)-C(10)	1.387(3)
C(9)-H(9)	0.91(2)
C(10)-C(11)	1.388(3)
C(10)-H(10)	0.92(3)
C(11)-C(12)	1.391(3)
C(11)-H(11)	0.97(2)
C(12)-H(12)	0.94(2)
C(13)-C(14)	1.373(3)
C(13)-H(13)	0.96(3)
C(14)-C(15)	1.389(3)
C(14)-H(14)	0.94(3)

C(15)-C(16)	1.401(3)
C(16)-C(17)	1.373(3)
C(16)-H(16)	0.94(3)
C(17)-H(17)	0.97(3)
C(18)-C(19)	1.496(3)
C(19)-C(20)	1.387(3)
C(19)-C(24)	1.390(3)
C(20)-C(21)	1.389(3)
C(20)-H(20)	0.93(3)
C(21)-C(22)	1.381(4)
C(21)-H(21)	0.99(3)
C(22)-C(23)	1.377(3)
C(22)-H(22)	0.94(3)
C(23)-C(24)	1.384(4)
C(23)-H(23)	0.97(3)
C(24)-H(24)	0.92(3)

N(1)#1-Ag(1)-N(1)	180.00(9)
N(3)#2-Ag(2)-N(3)	180.00(9)
O(4)-S(1)-O(3)	114.96(12)
O(4)-S(1)-O(5)	115.55(10)
O(3)-S(1)-O(5)	113.90(10)
O(4)-S(1)-C(25)	103.80(11)
O(3)-S(1)-C(25)	102.97(11)
O(5)-S(1)-C(25)	103.40(10)
C(1)-N(1)-C(5)	116.96(16)
C(1)-N(1)-Ag(1)	122.74(13)
C(5)-N(1)-Ag(1)	119.98(13)
C(6)-N(2)-C(3)	126.64(16)
C(6)-N(2)-H(2N)	117.4(16)
C(3)-N(2)-H(2N)	115.1(16)
C(17)-N(3)-C(13)	116.6(2)
C(17)-N(3)-Ag(2)	121.52(14)
C(13)-N(3)-Ag(2)	121.91(17)
C(18)-N(4)-C(15)	127.57(18)
C(18)-N(4)-H(4N)	118.1(17)

C(15)-N(4)-H(4N)	114.4(17)
N(1)-C(1)-C(2)	123.77(18)
N(1)-C(1)-H(1)	117.4(13)
C(2)-C(1)-H(1)	118.9(13)
C(1)-C(2)-C(3)	118.92(18)
C(1)-C(2)-H(2)	121.4(16)
C(3)-C(2)-H(2)	119.7(16)
C(2)-C(3)-C(4)	117.47(17)
C(2)-C(3)-N(2)	124.46(17)
C(4)-C(3)-N(2)	118.07(17)
C(5)-C(4)-C(3)	119.79(19)
C(5)-C(4)-H(3)	119.5(17)
C(3)-C(4)-H(3)	120.7(17)
N(1)-C(5)-C(4)	123.08(19)
N(1)-C(5)-H(4)	116.4(17)
C(4)-C(5)-H(4)	120.4(17)
O(1)-C(6)-N(2)	123.10(17)
O(1)-C(6)-C(7)	121.41(17)
N(2)-C(6)-C(7)	115.46(16)
C(12)-C(7)-C(8)	119.59(17)
C(12)-C(7)-C(6)	123.25(17)
C(8)-C(7)-C(6)	117.15(16)
C(9)-C(8)-C(7)	120.05(18)
C(9)-C(8)-H(8)	121.7(13)
C(7)-C(8)-H(8)	118.3(13)
C(8)-C(9)-C(10)	120.35(19)
C(8)-C(9)-H(9)	119.7(15)
C(10)-C(9)-H(9)	119.9(15)
C(9)-C(10)-C(11)	119.83(19)
C(9)-C(10)-H(10)	119.9(15)
C(11)-C(10)-H(10)	120.2(15)
C(10)-C(11)-C(12)	120.26(18)
C(10)-C(11)-H(11)	120.9(14)
C(12)-C(11)-H(11)	118.8(14)
C(11)-C(12)-C(7)	119.92(18)
C(11)-C(12)-H(12)	120.0(14)

C(7)-C(12)-H(12)	120.1(14)
N(3)-C(13)-C(14)	123.0(2)
N(3)-C(13)-H(13)	116.1(16)
C(14)-C(13)-H(13)	120.9(16)
C(13)-C(14)-C(15)	120.1(2)
C(13)-C(14)-H(14)	118.9(15)
C(15)-C(14)-H(14)	120.9(15)
C(14)-C(15)-N(4)	117.78(18)
C(14)-C(15)-C(16)	117.3(2)
N(4)-C(15)-C(16)	124.9(2)
C(17)-C(16)-C(15)	118.7(2)
C(17)-C(16)-H(16)	118.5(16)
C(15)-C(16)-H(16)	122.8(16)
N(3)-C(17)-C(16)	124.3(2)
N(3)-C(17)-H(17)	115.7(15)
C(16)-C(17)-H(17)	119.9(15)
O(2)-C(18)-N(4)	122.1(2)
O(2)-C(18)-C(19)	121.6(2)
N(4)-C(18)-C(19)	116.29(18)
C(20)-C(19)-C(24)	118.3(2)
C(20)-C(19)-C(18)	123.9(2)
C(24)-C(19)-C(18)	117.69(19)
C(19)-C(20)-C(21)	120.6(2)
C(19)-C(20)-H(20)	119.1(17)
C(21)-C(20)-H(20)	120.3(17)
C(22)-C(21)-C(20)	120.3(2)
C(22)-C(21)-H(21)	118.7(18)
C(20)-C(21)-H(21)	120.4(18)
C(23)-C(22)-C(21)	119.6(2)
C(23)-C(22)-H(22)	119.0(17)
C(21)-C(22)-H(22)	121.3(17)
C(22)-C(23)-C(24)	120.1(2)
C(22)-C(23)-H(23)	118.4(19)
C(24)-C(23)-H(23)	121.5(19)
C(23)-C(24)-C(19)	121.1(2)
C(23)-C(24)-H(24)	120.3(17)

C(19)-C(24)-H(24)	118.6(17)
F(2)-C(25)-F(1)	106.7(2)
F(2)-C(25)-F(3)	108.2(2)
F(1)-C(25)-F(3)	106.8(2)
F(2)-C(25)-S(1)	112.64(16)
F(1)-C(25)-S(1)	111.64(16)
F(3)-C(25)-S(1)	110.58(19)

Table S3: Bond lengths [Å] and angles [°] for (**4**).

Ag(1)-N(3)	2.127(3)
Ag(1)-N(1)	2.140(4)
P(1)-F(3)	1.532(4)
P(1)-F(4)	1.561(4)
P(1)-F(6)	1.579(4)
P(1)-F(2)	1.587(3)
P(1)-F(5)	1.605(4)
P(1)-F(1)	1.609(3)
O(1)-C(6)	1.219(5)
O(2)-C(18)	1.226(6)
N(1)-C(1)	1.339(6)
N(1)-C(5)	1.351(6)
N(2)-C(6)	1.380(6)
N(2)-C(3)	1.389(5)
N(2)-H(2N)	0.81(5)
N(3)-C(13)	1.345(6)
N(3)-C(17)	1.349(6)
N(4)-C(18)	1.371(7)
N(4)-C(15)	1.386(6)
N(4)-H(4N)	0.76(6)
C(1)-C(2)	1.366(7)
C(1)-H(1)	0.96(5)
C(2)-C(3)	1.394(6)
C(2)-H(2)	0.90(6)
C(3)-C(4)	1.393(7)

C(4)-C(5)	1.364(7)
C(4)-H(4)	0.88(5)
C(5)-H(5)	0.89(5)
C(6)-C(7)	1.493(5)
C(7)-C(8)	1.387(7)
C(7)-C(12)	1.391(6)
C(8)-C(9)	1.383(6)
C(8)-H(8)	0.93(5)
C(9)-C(10)	1.377(7)
C(9)-H(9)	0.99(6)
C(10)-C(11)	1.383(7)
C(10)-H(10)	0.92(5)
C(11)-C(12)	1.387(6)
C(11)-H(11)	0.87(5)
C(12)-H(12)	0.93(6)
C(13)-C(14)	1.377(6)
C(13)-H(13)	0.92(7)
C(14)-C(15)	1.396(7)
C(14)-H(14)	0.93(5)
C(15)-C(16)	1.392(6)
C(16)-C(17)	1.367(6)
C(16)-H(16)	0.93(6)
C(17)-H(17)	0.98(6)
C(18)-C(19)	1.482(6)
C(19)-C(20)	1.397(7)
C(19)-C(24)	1.401(7)
C(20)-C(21)	1.391(6)
C(20)-H(20)	0.88(5)
C(21)-C(22)	1.378(8)
C(21)-H(21)	0.87(6)
C(22)-C(23)	1.378(7)
C(22)-H(22)	0.91(5)
C(23)-C(24)	1.385(7)
C(23)-H(23)	0.93(7)
C(24)-H(24)	1.04(6)
O(3)-C(25)	1.358(10)

O(3)-H(3)	0.8400
C(25)-C(26)	1.460(14)
C(25)-H(25A)	0.9900
C(25)-H(25B)	0.9900
C(26)-C(25')	1.484(12)
C(26)-H(26A)	0.9900
C(26)-H(26B)	0.9900
C(25')-H(25C)	0.9800
C(25')-H(25D)	0.9800
C(25')-H(25E)	0.9800

N(3)-Ag(1)-N(1)	175.76(13)
F(3)-P(1)-F(4)	92.2(3)
F(3)-P(1)-F(6)	91.8(3)
F(4)-P(1)-F(6)	89.6(2)
F(3)-P(1)-F(2)	90.1(3)
F(4)-P(1)-F(2)	177.3(3)
F(6)-P(1)-F(2)	91.7(2)
F(3)-P(1)-F(5)	176.8(3)
F(4)-P(1)-F(5)	90.5(3)
F(6)-P(1)-F(5)	90.0(2)
F(2)-P(1)-F(5)	87.1(3)
F(3)-P(1)-F(1)	93.8(3)
F(4)-P(1)-F(1)	90.4(2)
F(6)-P(1)-F(1)	174.4(2)
F(2)-P(1)-F(1)	88.10(18)
F(5)-P(1)-F(1)	84.4(2)
C(1)-N(1)-C(5)	116.5(4)
C(1)-N(1)-Ag(1)	119.6(3)
C(5)-N(1)-Ag(1)	123.8(3)
C(6)-N(2)-C(3)	128.0(4)
C(6)-N(2)-H(2N)	118(4)
C(3)-N(2)-H(2N)	114(4)
C(13)-N(3)-C(17)	116.7(4)
C(13)-N(3)-Ag(1)	123.1(3)
C(17)-N(3)-Ag(1)	120.0(3)

C(18)-N(4)-C(15)	126.6(4)
C(18)-N(4)-H(4N)	116(5)
C(15)-N(4)-H(4N)	117(5)
N(1)-C(1)-C(2)	124.0(4)
N(1)-C(1)-H(1)	119(3)
C(2)-C(1)-H(1)	117(3)
C(1)-C(2)-C(3)	119.2(5)
C(1)-C(2)-H(2)	119(4)
C(3)-C(2)-H(2)	121(4)
N(2)-C(3)-C(4)	118.5(4)
N(2)-C(3)-C(2)	124.3(4)
C(4)-C(3)-C(2)	117.1(4)
C(5)-C(4)-C(3)	119.8(4)
C(5)-C(4)-H(4)	120(4)
C(3)-C(4)-H(4)	120(4)
N(1)-C(5)-C(4)	123.2(5)
N(1)-C(5)-H(5)	120(3)
C(4)-C(5)-H(5)	117(3)
O(1)-C(6)-N(2)	122.2(4)
O(1)-C(6)-C(7)	122.4(4)
N(2)-C(6)-C(7)	115.4(3)
C(8)-C(7)-C(12)	119.0(4)
C(8)-C(7)-C(6)	117.6(4)
C(12)-C(7)-C(6)	123.5(4)
C(9)-C(8)-C(7)	120.3(4)
C(9)-C(8)-H(8)	121(3)
C(7)-C(8)-H(8)	119(3)
C(10)-C(9)-C(8)	120.7(5)
C(10)-C(9)-H(9)	120(3)
C(8)-C(9)-H(9)	119(3)
C(9)-C(10)-C(11)	119.4(4)
C(9)-C(10)-H(10)	118(3)
C(11)-C(10)-H(10)	122(3)
C(10)-C(11)-C(12)	120.3(4)
C(10)-C(11)-H(11)	120(3)
C(12)-C(11)-H(11)	119(3)

C(11)-C(12)-C(7)	120.3(5)
C(11)-C(12)-H(12)	115(3)
C(7)-C(12)-H(12)	124(3)
N(3)-C(13)-C(14)	123.3(4)
N(3)-C(13)-H(13)	116(4)
C(14)-C(13)-H(13)	121(4)
C(13)-C(14)-C(15)	119.7(4)
C(13)-C(14)-H(14)	117(3)
C(15)-C(14)-H(14)	123(3)
N(4)-C(15)-C(16)	124.7(4)
N(4)-C(15)-C(14)	118.5(4)
C(16)-C(15)-C(14)	116.8(4)
C(17)-C(16)-C(15)	120.1(5)
C(17)-C(16)-H(16)	116(3)
C(15)-C(16)-H(16)	124(3)
N(3)-C(17)-C(16)	123.4(4)
N(3)-C(17)-H(17)	118(4)
C(16)-C(17)-H(17)	118(4)
O(2)-C(18)-N(4)	121.7(4)
O(2)-C(18)-C(19)	120.7(5)
N(4)-C(18)-C(19)	117.6(4)
C(20)-C(19)-C(24)	118.8(4)
C(20)-C(19)-C(18)	124.3(4)
C(24)-C(19)-C(18)	116.7(4)
C(21)-C(20)-C(19)	119.7(5)
C(21)-C(20)-H(20)	121(3)
C(19)-C(20)-H(20)	119(3)
C(22)-C(21)-C(20)	120.8(5)
C(22)-C(21)-H(21)	122(4)
C(20)-C(21)-H(21)	117(4)
C(21)-C(22)-C(23)	120.1(4)
C(21)-C(22)-H(22)	118(3)
C(23)-C(22)-H(22)	121(3)
C(22)-C(23)-C(24)	120.0(5)
C(22)-C(23)-H(23)	122(4)
C(24)-C(23)-H(23)	118(4)

C(23)-C(24)-C(19)	120.6(5)
C(23)-C(24)-H(24)	119(3)
C(19)-C(24)-H(24)	120(3)
C(25)-O(3)-H(3)	109.5
O(3)-C(25)-C(26)	118.2(8)
O(3)-C(25)-H(25A)	107.8
C(26)-C(25)-H(25A)	107.8
O(3)-C(25)-H(25B)	107.8
C(26)-C(25)-H(25B)	107.8
H(25A)-C(25)-H(25B)	107.1
C(25)-C(26)-C(25')	118.3(9)
C(25)-C(26)-H(26A)	107.7
C(25')-C(26)-H(26A)	107.7
C(25)-C(26)-H(26B)	107.7
C(25')-C(26)-H(26B)	107.7
H(26A)-C(26)-H(26B)	107.1
C(26)-C(25')-H(25C)	109.5
C(26)-C(25')-H(25D)	109.5
H(25C)-C(25')-H(25D)	109.5
C(26)-C(25')-H(25E)	109.5
H(25C)-C(25')-H(25E)	109.5
H(25D)-C(25')-H(25E)	109.5

Table S4: Bond lengths [Å] and angles [°] for (**5)**.

Ag(1)-N(1)	2.123(2)
Ag(1)-N(3)	2.125(2)
O(1)-C(6)	1.223(3)
O(2)-C(18)	1.228(3)
N(1)-C(1)	1.343(3)
N(1)-C(5)	1.345(3)
N(2)-C(6)	1.371(3)
N(2)-C(3)	1.391(3)
N(2)-H(2N)	0.78(3)
N(3)-C(17)	1.345(3)

N(3)-C(13)	1.349(3)
N(4)-C(18)	1.375(3)
N(4)-C(15)	1.394(3)
N(4)-H(4N)	0.79(3)
C(1)-C(2)	1.379(3)
C(1)-H(1)	1.00(3)
C(2)-C(3)	1.395(3)
C(2)-H(2)	0.90(3)
C(3)-C(4)	1.394(3)
C(4)-C(5)	1.374(3)
C(4)-H(4)	0.89(3)
C(5)-H(5)	0.89(3)
C(6)-C(7)	1.497(3)
C(7)-C(12)	1.390(3)
C(7)-C(8)	1.395(3)
C(8)-C(9)	1.386(3)
C(8)-H(8)	0.95(3)
C(9)-C(10)	1.387(4)
C(9)-H(9)	0.91(3)
C(10)-C(11)	1.387(4)
C(10)-H(10)	0.92(3)
C(11)-C(12)	1.389(4)
C(11)-H(11)	0.94(3)
C(12)-H(12)	0.92(3)
C(13)-C(14)	1.376(3)
C(13)-H(13)	0.94(3)
C(14)-C(15)	1.394(3)
C(14)-H(14)	0.87(3)
C(15)-C(16)	1.398(3)
C(16)-C(17)	1.378(3)
C(16)-H(16)	0.86(3)
C(17)-H(17)	0.96(3)
C(18)-C(19)	1.497(3)
C(19)-C(20)	1.394(3)
C(19)-C(24)	1.395(3)
C(20)-C(21)	1.389(3)

C(20)-H(20)	0.93(3)
C(21)-C(22)	1.386(4)
C(21)-H(21)	0.95(3)
C(22)-C(23)	1.384(4)
C(22)-H(22)	1.02(3)
C(23)-C(24)	1.383(3)
C(23)-H(23)	0.91(3)
C(24)-H(24)	0.94(3)
B(1)-F(4A)	1.236(8)
B(1)-F(2A)	1.330(14)
B(1)-F(3)	1.346(4)
B(1)-F(2)	1.350(4)
B(1)-F(1A)	1.394(17)
B(1)-F(1)	1.396(4)
B(1)-F(4)	1.436(4)
B(1)-F(3A)	1.550(10)
C(25)-O(3)	1.152(10)
C(25)-C(25)#1	1.502(11)
C(25A)-O(3A)	1.12(2)
C(25A)-C(25A)#1	1.61(3)

N(1)-Ag(1)-N(3)	178.12(7)
C(1)-N(1)-C(5)	116.9(2)
C(1)-N(1)-Ag(1)	121.18(16)
C(5)-N(1)-Ag(1)	121.81(16)
C(6)-N(2)-C(3)	126.9(2)
C(6)-N(2)-H(2N)	117(2)
C(3)-N(2)-H(2N)	117(2)
C(17)-N(3)-C(13)	117.0(2)
C(17)-N(3)-Ag(1)	120.21(16)
C(13)-N(3)-Ag(1)	122.77(16)
C(18)-N(4)-C(15)	127.8(2)
C(18)-N(4)-H(4N)	116(2)
C(15)-N(4)-H(4N)	116(2)
N(1)-C(1)-C(2)	124.1(2)
N(1)-C(1)-H(1)	117.1(17)

C(2)-C(1)-H(1)	118.8(17)
C(1)-C(2)-C(3)	118.6(2)
C(1)-C(2)-H(2)	117(2)
C(3)-C(2)-H(2)	125(2)
N(2)-C(3)-C(4)	118.1(2)
N(2)-C(3)-C(2)	124.3(2)
C(4)-C(3)-C(2)	117.6(2)
C(5)-C(4)-C(3)	119.8(2)
C(5)-C(4)-H(4)	118(2)
C(3)-C(4)-H(4)	122(2)
N(1)-C(5)-C(4)	123.0(2)
N(1)-C(5)-H(5)	117.9(18)
C(4)-C(5)-H(5)	119.0(18)
O(1)-C(6)-N(2)	122.7(2)
O(1)-C(6)-C(7)	121.3(2)
N(2)-C(6)-C(7)	116.0(2)
C(12)-C(7)-C(8)	119.6(2)
C(12)-C(7)-C(6)	123.7(2)
C(8)-C(7)-C(6)	116.7(2)
C(9)-C(8)-C(7)	120.5(2)
C(9)-C(8)-H(8)	119.5(19)
C(7)-C(8)-H(8)	120.0(19)
C(8)-C(9)-C(10)	119.7(2)
C(8)-C(9)-H(9)	121.1(19)
C(10)-C(9)-H(9)	119.1(19)
C(11)-C(10)-C(9)	120.0(2)
C(11)-C(10)-H(10)	120.3(19)
C(9)-C(10)-H(10)	119.7(19)
C(10)-C(11)-C(12)	120.5(2)
C(10)-C(11)-H(11)	122(2)
C(12)-C(11)-H(11)	117(2)
C(11)-C(12)-C(7)	119.7(2)
C(11)-C(12)-H(12)	120.6(19)
C(7)-C(12)-H(12)	119.6(19)
N(3)-C(13)-C(14)	123.0(2)
N(3)-C(13)-H(13)	117.0(18)

C(14)-C(13)-H(13)	120.0(18)
C(13)-C(14)-C(15)	120.0(2)
C(13)-C(14)-H(14)	120(2)
C(15)-C(14)-H(14)	120(2)
C(14)-C(15)-N(4)	118.7(2)
C(14)-C(15)-C(16)	117.1(2)
N(4)-C(15)-C(16)	124.2(2)
C(17)-C(16)-C(15)	119.3(2)
C(17)-C(16)-H(16)	118(2)
C(15)-C(16)-H(16)	123(2)
N(3)-C(17)-C(16)	123.6(2)
N(3)-C(17)-H(17)	118.2(19)
C(16)-C(17)-H(17)	118.2(19)
O(2)-C(18)-N(4)	122.7(2)
O(2)-C(18)-C(19)	121.4(2)
N(4)-C(18)-C(19)	115.86(19)
C(20)-C(19)-C(24)	119.3(2)
C(20)-C(19)-C(18)	123.6(2)
C(24)-C(19)-C(18)	117.0(2)
C(21)-C(20)-C(19)	119.8(2)
C(21)-C(20)-H(20)	121.3(17)
C(19)-C(20)-H(20)	118.8(17)
C(22)-C(21)-C(20)	120.6(2)
C(22)-C(21)-H(21)	121.0(19)
C(20)-C(21)-H(21)	118.3(19)
C(23)-C(22)-C(21)	119.6(2)
C(23)-C(22)-H(22)	122.7(17)
C(21)-C(22)-H(22)	117.7(17)
C(24)-C(23)-C(22)	120.5(2)
C(24)-C(23)-H(23)	116.9(19)
C(22)-C(23)-H(23)	122.7(19)
C(23)-C(24)-C(19)	120.3(2)
C(23)-C(24)-H(24)	122(2)
C(19)-C(24)-H(24)	118(2)
F(4A)-B(1)-F(2A)	121.9(7)
F(4A)-B(1)-F(3)	52.9(4)

F(2A)-B(1)-F(3)	141.8(6)
F(4A)-B(1)-F(2)	123.5(4)
F(2A)-B(1)-F(2)	27.5(5)
F(3)-B(1)-F(2)	117.4(4)
F(4A)-B(1)-F(1A)	125.6(9)
F(2A)-B(1)-F(1A)	110.1(10)
F(3)-B(1)-F(1A)	95.0(7)
F(2)-B(1)-F(1A)	109.8(8)
F(4A)-B(1)-F(1)	120.7(4)
F(2A)-B(1)-F(1)	103.9(6)
F(3)-B(1)-F(1)	109.5(3)
F(2)-B(1)-F(1)	114.6(3)
F(1A)-B(1)-F(1)	22.4(6)
F(4A)-B(1)-F(4)	48.9(4)
F(2A)-B(1)-F(4)	86.7(6)
F(3)-B(1)-F(4)	101.7(3)
F(2)-B(1)-F(4)	107.0(3)
F(1A)-B(1)-F(4)	126.0(7)
F(1)-B(1)-F(4)	105.2(3)
F(4A)-B(1)-F(3A)	98.3(6)
F(2A)-B(1)-F(3A)	101.1(7)
F(3)-B(1)-F(3A)	51.1(4)
F(2)-B(1)-F(3A)	73.7(5)
F(1A)-B(1)-F(3A)	86.0(8)
F(1)-B(1)-F(3A)	108.4(5)
F(4)-B(1)-F(3A)	142.4(5)
O(3)-C(25)-C(25)#1	119.5(8)
O(3A)-C(25A)-C(25A)#1	134(2)

Table S5. Bond lengths [Å] and angles [°] for **6**.

Ag(1)-N(3)	2.1153(14)
Ag(1)-N(1)	2.1171(15)
S(1)-O(5)	1.4400(13)
S(1)-O(3)	1.4602(13)

S(1)-O(4)	1.4668(14)
S(1)-C(25)	1.7738(17)
O(1)-C(6)	1.216(2)
O(2)-C(18)	1.218(2)
N(1)-C(1)	1.342(2)
N(1)-C(5)	1.349(2)
N(2)-C(6)	1.382(2)
N(2)-C(3)	1.390(2)
N(2)-H(2N)	0.83(2)
N(3)-C(17)	1.341(2)
N(3)-C(13)	1.344(2)
N(4)-C(18)	1.374(2)
N(4)-C(15)	1.390(2)
N(4)-H(4N)	0.82(2)
C(1)-C(2)	1.374(3)
C(1)-H(1)	0.94(2)
C(2)-C(3)	1.399(3)
C(2)-H(2)	0.89(2)
C(3)-C(4)	1.394(2)
C(4)-C(5)	1.377(3)
C(4)-H(4)	0.92(2)
C(5)-H(5)	0.96(2)
C(6)-C(7)	1.496(2)
C(7)-C(8)	1.391(3)
C(7)-C(12)	1.394(3)
C(8)-C(9)	1.389(3)
C(8)-H(8)	0.96(2)
C(9)-C(10)	1.381(3)
C(9)-H(9)	0.94(2)
C(10)-C(11)	1.386(3)
C(10)-H(10)	0.95(3)
C(11)-C(12)	1.382(3)
C(11)-H(11)	0.96(3)
C(12)-H(12)	0.94(2)
C(13)-C(14)	1.378(3)
C(13)-H(13)	0.93(2)

C(14)-C(15)	1.392(2)
C(14)-H(14)	0.98(3)
C(15)-C(16)	1.396(2)
C(16)-C(17)	1.368(2)
C(16)-H(16)	0.93(2)
C(17)-H(17)	0.92(2)
C(18)-C(19)	1.498(2)
C(19)-C(24)	1.393(3)
C(19)-C(20)	1.396(2)
C(20)-C(21)	1.383(3)
C(20)-H(20)	0.96(2)
C(21)-C(22)	1.383(3)
C(21)-H(21)	0.95(2)
C(22)-C(23)	1.385(3)
C(22)-H(22)	0.90(2)
C(23)-C(24)	1.389(3)
C(23)-H(23)	0.97(2)
C(24)-H(24)	0.92(2)
C(25)-C(30)	1.383(3)
C(25)-C(26)	1.389(3)
C(26)-C(27)	1.386(3)
C(26)-H(26)	0.93(2)
C(27)-C(28)	1.388(4)
C(27)-H(27)	0.90(3)
C(28)-C(29)	1.377(4)
C(28)-C(31)	1.519(3)
C(29)-C(30)	1.394(3)
C(29)-H(29)	0.94(3)
C(30)-H(30)	0.93(2)
C(31)-H(31A)	0.9800
C(31)-H(31B)	0.9800
C(31)-H(31C)	0.9800

N(3)-Ag(1)-N(1)	176.86(5)
O(5)-S(1)-O(3)	113.68(9)
O(5)-S(1)-O(4)	113.40(8)

O(3)-S(1)-O(4)	110.78(8)
O(5)-S(1)-C(25)	107.07(8)
O(3)-S(1)-C(25)	105.79(8)
O(4)-S(1)-C(25)	105.40(8)
C(1)-N(1)-C(5)	116.87(15)
C(1)-N(1)-Ag(1)	122.83(13)
C(5)-N(1)-Ag(1)	120.29(12)
C(6)-N(2)-C(3)	127.58(15)
C(6)-N(2)-H(2N)	115.8(15)
C(3)-N(2)-H(2N)	116.3(15)
C(17)-N(3)-C(13)	116.73(15)
C(17)-N(3)-Ag(1)	119.09(12)
C(13)-N(3)-Ag(1)	124.09(12)
C(18)-N(4)-C(15)	127.57(15)
C(18)-N(4)-H(4N)	118.8(16)
C(15)-N(4)-H(4N)	113.6(16)
N(1)-C(1)-C(2)	123.44(18)
N(1)-C(1)-H(1)	117.8(13)
C(2)-C(1)-H(1)	118.8(13)
C(1)-C(2)-C(3)	119.65(17)
C(1)-C(2)-H(2)	121.8(14)
C(3)-C(2)-H(2)	118.6(14)
N(2)-C(3)-C(4)	125.22(16)
N(2)-C(3)-C(2)	117.65(15)
C(4)-C(3)-C(2)	117.13(16)
C(5)-C(4)-C(3)	119.43(18)
C(5)-C(4)-H(4)	118.5(15)
C(3)-C(4)-H(4)	122.1(15)
N(1)-C(5)-C(4)	123.47(17)
N(1)-C(5)-H(5)	117.5(14)
C(4)-C(5)-H(5)	118.9(14)
O(1)-C(6)-N(2)	123.64(17)
O(1)-C(6)-C(7)	122.12(16)
N(2)-C(6)-C(7)	114.23(15)
C(8)-C(7)-C(12)	119.44(17)
C(8)-C(7)-C(6)	122.40(16)

C(12)-C(7)-C(6)	118.11(16)
C(9)-C(8)-C(7)	120.09(18)
C(9)-C(8)-H(8)	118.1(13)
C(7)-C(8)-H(8)	121.8(13)
C(10)-C(9)-C(8)	119.97(19)
C(10)-C(9)-H(9)	121.2(15)
C(8)-C(9)-H(9)	118.8(15)
C(9)-C(10)-C(11)	120.23(19)
C(9)-C(10)-H(10)	118.1(16)
C(11)-C(10)-H(10)	121.6(16)
C(12)-C(11)-C(10)	120.07(19)
C(12)-C(11)-H(11)	119.2(14)
C(10)-C(11)-H(11)	120.6(14)
C(11)-C(12)-C(7)	120.17(18)
C(11)-C(12)-H(12)	120.0(15)
C(7)-C(12)-H(12)	119.8(15)
N(3)-C(13)-C(14)	123.54(17)
N(3)-C(13)-H(13)	117.1(14)
C(14)-C(13)-H(13)	119.3(14)
C(13)-C(14)-C(15)	119.33(17)
C(13)-C(14)-H(14)	120.1(15)
C(15)-C(14)-H(14)	120.6(15)
N(4)-C(15)-C(14)	125.17(16)
N(4)-C(15)-C(16)	117.76(15)
C(14)-C(15)-C(16)	117.06(16)
C(17)-C(16)-C(15)	119.80(16)
C(17)-C(16)-H(16)	120.2(14)
C(15)-C(16)-H(16)	119.9(14)
N(3)-C(17)-C(16)	123.52(17)
N(3)-C(17)-H(17)	117.7(14)
C(16)-C(17)-H(17)	118.7(14)
O(2)-C(18)-N(4)	122.63(16)
O(2)-C(18)-C(19)	120.95(16)
N(4)-C(18)-C(19)	116.42(14)
C(24)-C(19)-C(20)	118.96(16)
C(24)-C(19)-C(18)	124.05(16)

C(20)-C(19)-C(18)	116.95(15)
C(21)-C(20)-C(19)	120.49(17)
C(21)-C(20)-H(20)	120.2(13)
C(19)-C(20)-H(20)	119.3(13)
C(22)-C(21)-C(20)	120.36(17)
C(22)-C(21)-H(21)	119.7(14)
C(20)-C(21)-H(21)	119.9(14)
C(21)-C(22)-C(23)	119.54(17)
C(21)-C(22)-H(22)	119.5(14)
C(23)-C(22)-H(22)	120.9(14)
C(22)-C(23)-C(24)	120.56(18)
C(22)-C(23)-H(23)	122.4(15)
C(24)-C(23)-H(23)	117.1(15)
C(23)-C(24)-C(19)	120.09(18)
C(23)-C(24)-H(24)	119.5(14)
C(19)-C(24)-H(24)	120.4(14)
C(30)-C(25)-C(26)	120.56(17)
C(30)-C(25)-S(1)	120.31(14)
C(26)-C(25)-S(1)	119.10(14)
C(27)-C(26)-C(25)	119.1(2)
C(27)-C(26)-H(26)	119.9(15)
C(25)-C(26)-H(26)	121.0(15)
C(26)-C(27)-C(28)	121.4(2)
C(26)-C(27)-H(27)	115.3(18)
C(28)-C(27)-H(27)	123.2(17)
C(29)-C(28)-C(27)	118.47(19)
C(29)-C(28)-C(31)	121.0(3)
C(27)-C(28)-C(31)	120.5(3)
C(28)-C(29)-C(30)	121.5(2)
C(28)-C(29)-H(29)	122.5(16)
C(30)-C(29)-H(29)	116.0(16)
C(25)-C(30)-C(29)	119.0(2)
C(25)-C(30)-H(30)	119.3(13)
C(29)-C(30)-H(30)	121.7(13)
C(28)-C(31)-H(31A)	109.5
C(28)-C(31)-H(31B)	109.5

H(31A)-C(31)-H(31B)	109.5
C(28)-C(31)-H(31C)	109.5
H(31A)-C(31)-H(31C)	109.5
H(31B)-C(31)-H(31C)	109.5
